# Azithromycin Exposure Induces Transient Microbial Composition Shifts and Decreases the Airway Microbiota Resilience from Outdoor PM_2.5_ Stress in Healthy Adults: a Randomized, Double-Blind, Placebo-Controlled Trial

**DOI:** 10.1128/spectrum.02066-22

**Published:** 2023-04-24

**Authors:** Sisi Du, Lianhan Shang, Xiaohui Zou, Xiaoyan Deng, Aihua Sun, Shengrui Mu, Jiankang Zhao, Yimin Wang, Xiaoxuan Feng, Binbin Li, Chunlei Wang, Shuai Liu, Binghuai Lu, Yingmei Liu, Rongrong Zhang, Yigang Tong, Bin Cao

**Affiliations:** a China-Japan Friendship Hospital, National Clinical Research Center for Respiratory Diseases, National Center for Respiratory Medicine, Clinical Center for Pulmonary Infections, Capital Medical University, Beijing, China; b Institute of Respiratory Medicine, Chinese Academy of Medical Sciences, Peking Union Medical College, Beijing, China; c Department of Pulmonary and Critical Care Medicine, Center for Respiratory Diseases, China-Japan Friendship Hospital, Beijing, China; d Tsinghua University-Peking University Joint Center for Life Sciences, Beijing, China; e Department of Pulmonary and Critical Care Medicine, Jin Yin-tan Hospital, Wuhan, Hubei, China; f State Key Laboratory of Pathogen and Biosecurity, Beijing Institute of Microbiology and Epidemiology, Beijing, China; g College of Information Science and Technology, Beijing University of Chemical Technology, Beijing, China; h Changping Laboratory, Beijing, China; Huazhong University of Science and Technology

**Keywords:** azithromycin, airway microbiota, healthy volunteers, biodiversity, stability, PM2.5, network

## Abstract

Inappropriate antibiotic prescriptions are common for patients with upper respiratory tract infections (URTIs). Few data exist regarding the effects of antibiotic administration on airway microbiota among healthy adults. We conducted a randomized, double-blind, placebo-controlled trial to characterize the airway microbiota longitudinally in healthy adults using 16S rRNA gene sequencing and quantification. Both the induced sputum and oral wash samples were collected over a 60-day period following a 3-day intervention with 500 mg azithromycin or placebo. Environmental information, including air quality data (particulate matter [PM_2.5_] and PM_10_, air quality index [AQI] values), were also collected during the study. A total of 48 healthy volunteers were enrolled and randomly assigned into two groups. Azithromycin did not alter bacterial load but significantly reduced species richness and Shannon index. Azithromycin exposure resulted in a decrease in the detection rate and relative abundance of different genera belonging to *Veillonellaceae*, Leptotrichia, Fusobacterium, Neisseria, and Haemophilus. In contrast, the relative abundance of taxa belonging to Streptococcus increased immediately after azithromycin intervention. The shifts in the diversity of the microbiology composition took between 14 and 60 days to recover, depending on the measure used: either UniFrac phylogenetic distance or α-diversity. Outdoor environmental perturbations, especially the high concentration of PM_2.5_, contributed to novel variability in microbial community composition of the azithromycin group at D30 (30 days after baseline). The network analysis found that azithromycin altered the microbial interactions within airway microbiota. The influence was still obvious at D14 when the relative abundance of most taxa had returned to the baseline level. Compared to the sputum microbiota, oral cavity microbiota had a different pattern of change over time. The induced sputum microbial data can represent the airway microbiota composition in healthy adults. Azithromycin may have transient effects in the airway microbiota of healthy adults and decrease the airway microbiota resilience against outdoor environmental stress. The influence of azithromycin on microbial interactions is noteworthy, although the airway microbiota has returned to a near-baseline level.

**IMPORTANCE** The influence of antibiotic administration on the airway microbiota of healthy adults remains unknown. This study is a randomized, double-blind, placebo-controlled trial aiming to investigate the microbial shifts in airways after exposure to azithromycin among heathy adults. We find that azithromycin changes the airway microbial community composition of healthy adults and decreases the airway microbiota resilience against outdoor environmental stress. This study depicts the longitudinal recovery trajectory of airway microbiota after the antibiotic perturbation and may provide reference for appropriate antibiotic prescription.

## INTRODUCTION

Human airway microbiota forms a complex, balanced ecosystem and serves as the gatekeeper for respiratory health (1). Both animal models and human studies have demonstrated that the respiratory microbiota is related to the maturation of alveolar architecture and shapes the local immune system (2–5). Among the healthy children, it has been shown that early-life healthy airway microbiota development enforces the defense against respiratory tract infections (6). In addition, the respiratory microbiota may have a role in dealing with inhaled pollutants (7). The dysbiosis of airway microbiota, driven by decreased microbial diversity and overgrowth of certain bacterium, may contribute to the occurrence and development of respiratory diseases (8, 9).

Globally, inappropriate antibiotic prescribing is common, and the majority of such prescriptions are for patients with upper respiratory tract infections (URTIs), which are self-limiting and of viral origin in most cases (10, 11). For URTI patients with viral etiology, antibiotics may not provide clinical benefits and may lead to adverse events due to disturbance of microbiota, such as Clostridium difficile infections (12). In addition, antibiotic use is considered to be one of the most important perturbations for the airway microbiota. A 7-day antibiotic course was shown to alter the lung microbiota in healthy murine (3). However, few data exist regarding the variation in airway microbiota after antibiotic administration among healthy adults, as well as the longitudinal description about the recovery trajectory of microbiota shifts after the antibiotic perturbation.

Azithromycin, a macrolide antibiotic, is widely used in URTIs because of the broad antibacterial spectrum, including Gram-positive bacteria (Streptococcus pneumoniae, Streptococcus pyogenes, etc.), Gram-negative bacteria (Neisseria gonorrhea, Haemophilus influenzae, etc.), and atypical pathogens (Mycoplasma pneumoniae, Chlamydia spp., and Legionella pneumophila, etc.) (13). In addition, azithromycin shows potential effects on viral infections, such as influenza and chronic respiratory infections due to its antiviral and anti-inflammatory activities (14, 15). In order to fully depict antibiotics’ effects on human microbiota, it is important to understand the effects among healthy individuals, which may provide reference for appropriate antibiotic prescription.

The exposure to elevated levels of outdoor PM_2.5_ contributes to changes in sputum and pharyngeal microbiota composition, even in healthy individuals (16, 17). Mouse model studies demonstrate that PM_2.5_ exposure alters the lung microbiota composition, which affects pulmonary inflammation and the metabolic profile of hosts (18–21). Li et al. (22) find that the air pollution is related to the respiratory tract microbiota among healthy volunteers in China. Therefore, it is also significant to identify the effects of outdoor environmental factors on microbiota after antibiotic exposure.

In this randomized controlled study, we focused on the airway microbiota dynamic variation in the healthy adults. The 16S rRNA gene sequencing was applied to the induced sputum samples collected before administration of the drugs (3-day azithromycin and placebo) and at 4 time points over 60 days postdosing. We aim to track the process of variation and re-establishment of biodiversity, microbial interactions, and community composition of the airway microbiota after a 3-day azithromycin administration. We also estimated the potential influence of outdoor environmental factors (e.g., humidity, concentration of PM_2.5_ and PM_10_) on airway microbiota during the follow-up.

## RESULTS

### Baseline characteristics.

**FIG 1:**
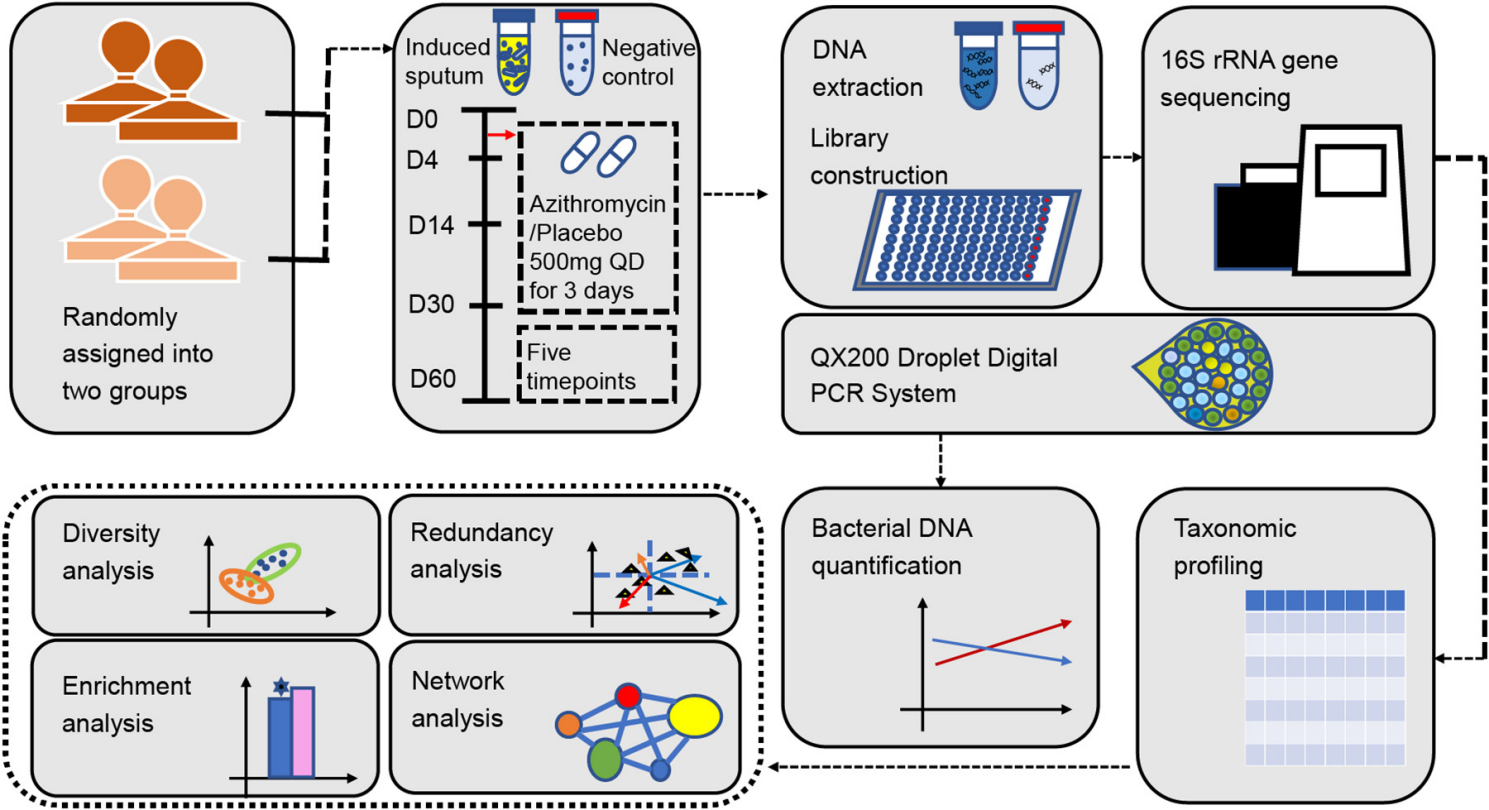
Overview of the study design and sample collection. The healthy volunteers (*n* = 48) enrolled in this study were stratified by gender and randomly assigned to either the azithromycin or the placebo group (1:1). Either 500 mg azithromycin or 500 mg starch contained in identical opaque white capsules was administered once daily (QD) for 3 days. The sputum samples were collected the day before the drug administration (D0), the day after the treatment course is completed (D4), and at 14 (D14), 30 (D30), and 60 days (D60) postdosing. Then 16S rRNA gene sequencing was applied to the induced sputum samples and negative-control samples. The bacterial DNA of sputum samples was quantified using a QX200 Droplet Digital PCR system. The microbial ecology analysis is performed on the data produced from the study later.

From October 8, 2018, to October 22, 2018, we recruited 48 healthy volunteers with an average age of 26.6 years (Table 1). Participants underwent follow-up visits from October 2018 to December 2018 (Fig. 1). In total, 221 induced sputum samples were collected (Table 2). All the healthy volunteers resided in Beijing, China, and never left Beijing during the study period. Thirty-eight subjects (79.2%) completed the five time points. One subject was diagnosed with acute laryngitis after time point D0. Six subjects had colds at D30, and three had colds at D60. All dropouts were lost to follow-up right after the last visit. The induced sputum samples were analyzed by sequencing of the 16S rRNA genes, yielding 23,693,658 reads after quality filtering with the median reads, 69,349 per sample. Since the consumables and reagents used in the experiment procedures (dithiothreitol [DTT], DNA isolation and library preparation, etc.) might contain bacterial DNA, it was important to identify the impact of contamination on sequencing results. Thus, the 36 experimental controls were also sequenced alongside the sputum samples obtaining 4,152 reads/sample.

**TABLE 1:** Demographics of healthy volunteers at baseline^*a*^

Parameter	Azithromycin group (*n* = 24)	Placebo group (*n* = 24)	*P* value
Male sex, *n* (%)	12 (50)	12 (50)	
Age, yr, median (IQR)	25 (3.00)	25 (3.25)	0.60^*e*^
ht, cm, mean ± SD	168.75 ± 7.57	167.21 ± 8.52	0.51^*f*^
wt, kg, mean ± SD	62.71 ± 12.27	61.27 ± 9.55	0.65^*f*^
Race, *n* (%)			
Han	23 (95.83)	22 (91.67)	1^*g*^
Others	1 (4.17)	2 (8.33)	
Residence^*b*^, *n* (%)			
Chaoyang district	20 (83.33)	19 (79.17)	1^*g*^
Others	4 (16.67)	5 (20.83)	
Occupation, *n* (%)			
Student’s	15 (62.5)	19 (79.17)	0.20^*h*^
Others^*c*^	9 (37.5)	5 (20.83)	
Microbial diversity^*d*^ (D0)			
α-Diversity, mean ± SD			
Richness	418.62 ± 89.73	414.88 ± 88.94	0.88^*f*^
Shannon	6.01 ± 0.41	6.02 ± 0.33	0.12^*f*^
β-Diversity			
Unweighted UniFrac distance	NA	NA	0.76^*i*^
Weighted UniFrac distance	NA	NA	0.76^*i*^

a IQR, interquartile range; NA, not applicable; SD, standard deviation.

b All the participants resided in Beijing, China. Most of them lived in the Chaoyang district, while others lived in the Changping, Shijingshan, Haidian, Dongcheng, and Fengtai districts.

c Other occupations included physicians, company employees, and civil servants.

d Diversity of the baseline sputum microbiota of the healthy volunteers using samples collected before drug administration.

e Compared by two-sided Wilcoxon rank sum test.

f Compared by two-sided Student’s *t* test.

g Compared by two-sided chi-squared test with continuity correction.

h Compared by two-sided Pearson’s chi-squared test.

i Compared by permutational multivariate analysis of variance (PERMANOVA).

**TABLE 2:** Data of sputum samples collected at different time points^*a*^

Time points	Azithromycin group (*n* = 114)	Placebo group (*n* = 107)
D0	24	24
D4	24	23
D14	24	23
D30	21	20
D60	21	17

a The sputum samples were collected before the day of drug administration (D0), the day after the treatment course was completed (D4), and at 14 days (D14), 30 days (D30), and 60 days (D60) postdosing. A total of 38 of all the subjects completed the five time points. Six subjects had a cold at time point D30. Three subjects had common cold at time point D60. One subject was diagnosed with acute laryngitis after time point D0. All dropouts were lost to follow-up right after the last visit.

### Minimal influence of procedural contaminations on sputum sample sequencing.

Based on both unweighted UniFrac and weighted UniFrac distance, the difference in microbial community composition between sputum samples and experimental control samples was significant (permutational multivariate analysis of variance [PERMANOVA], *R*^2^ = 0.35 and 0.28; *P* = 0.001 and 0.001; Fig. S1a to b). The five zero-radius operational taxonomic units (ZOTUs, 100% sequence similarity and denoised by UNOISE3) detected in sputum samples with the highest abundance comprised 23.27% of all sequences, while comprising 7.63% of all sequences detected in control samples (Fig. S1c). The most abundant ZOTU detected in control specimens (ZOTU36) accounted for only 0.03% of all sequences detected in sputum specimens (Fig. S1d). We performed microbial ecology analysis using the decontam package, the function of which was to identify contaminants in sequencing data based on statistical approach (23). We used both the frequency and prevalence methods with threshold 0.1 and 0.5, respectively, and confirmed only 3 contaminants ZOTUs (ZOTU1757, ZOTU2042, and ZOTU3065) in our data (Fig. S1e to f; Table S1). The relative abundance of the three ZOTUs were very low in both sputum and negative-control samples (comprising 6.33 × 10^−5^%, 1.98 × 10^−4^%, and 4.64 × 10^−5^% of all sputum samples sequences and 0.031%, 1.34 × 10^−3^%, and 1.34 × 10^−2^% of all negative-control samples sequences). ZOTU1757, ZOTU2042, and ZOTU3065 appeared in only 8 (3.62%), 5 (2.26%), and 4 (1.81%) of the sputum samples and 3 (8.33%), 1 (2.78%), and 1 (2.78%) of the control samples, respectively. The rare and low-prevalence sequences often had no effect on the microbial diversity (24). In summary, we found little evidence of procedural contaminations influencing the species detected in sputum and removed the three ZOTUs from the final analysis.

### The bacterial DNA load was not significantly altered in healthy volunteers’ airways after azithromycin administration.

**FIG 2:**
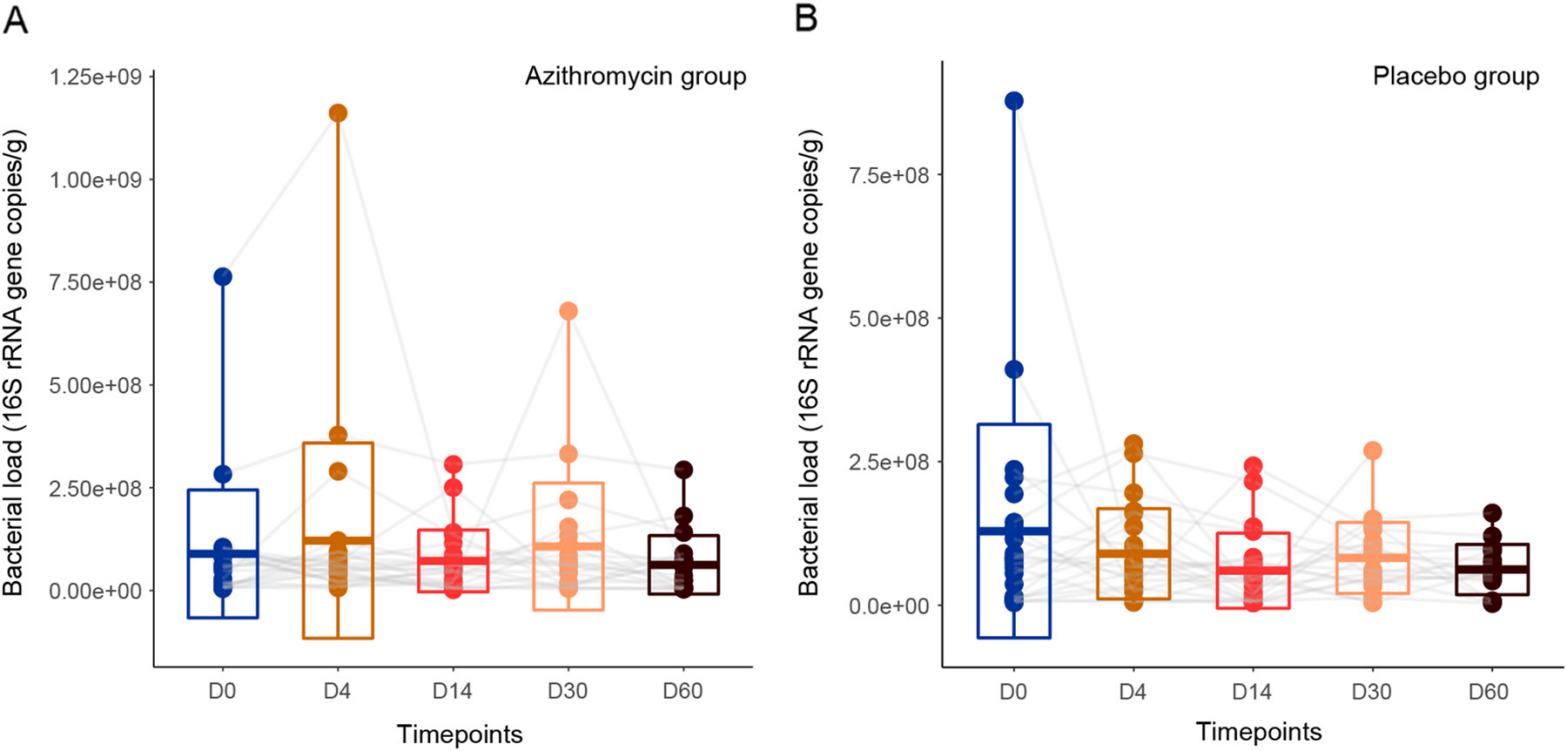
Sputum bacterial DNA quantification using droplet digital PCR of the 16S rRNA gene. There was no significant difference in sputum bacterial DNA load across different time points in either the azithromycin group (A) or the placebo group (B) (Wilcoxon signed-rank test, Bonferroni adjusted *P* values [*q*] > 0.05 for all). The boxplots represent the bacterial DNA load for the subjects (center line, mean; box limits, ±standard deviation; whisker limits, maximum/minimum). The points are connected across time points by gray lines.

We quantified the bacterial DNA of every sputum sample using droplet digital PCR system. There was considerable variation across individual sputum samples (first quartile and third quartile, 1.8 × 10^7^ to 9.7 × 10^7^ copies/g). However, we found no significant difference in sputum bacterial DNA load across different time points in either azithromycin or placebo group (Wilcoxon signed-rank test, Bonferroni adjusted *P* values [*q*] > 0.05 for all; Fig. 2).

### Shifts in sputum phylogenetic diversity after antibiotic exposure.

**FIG 3:**
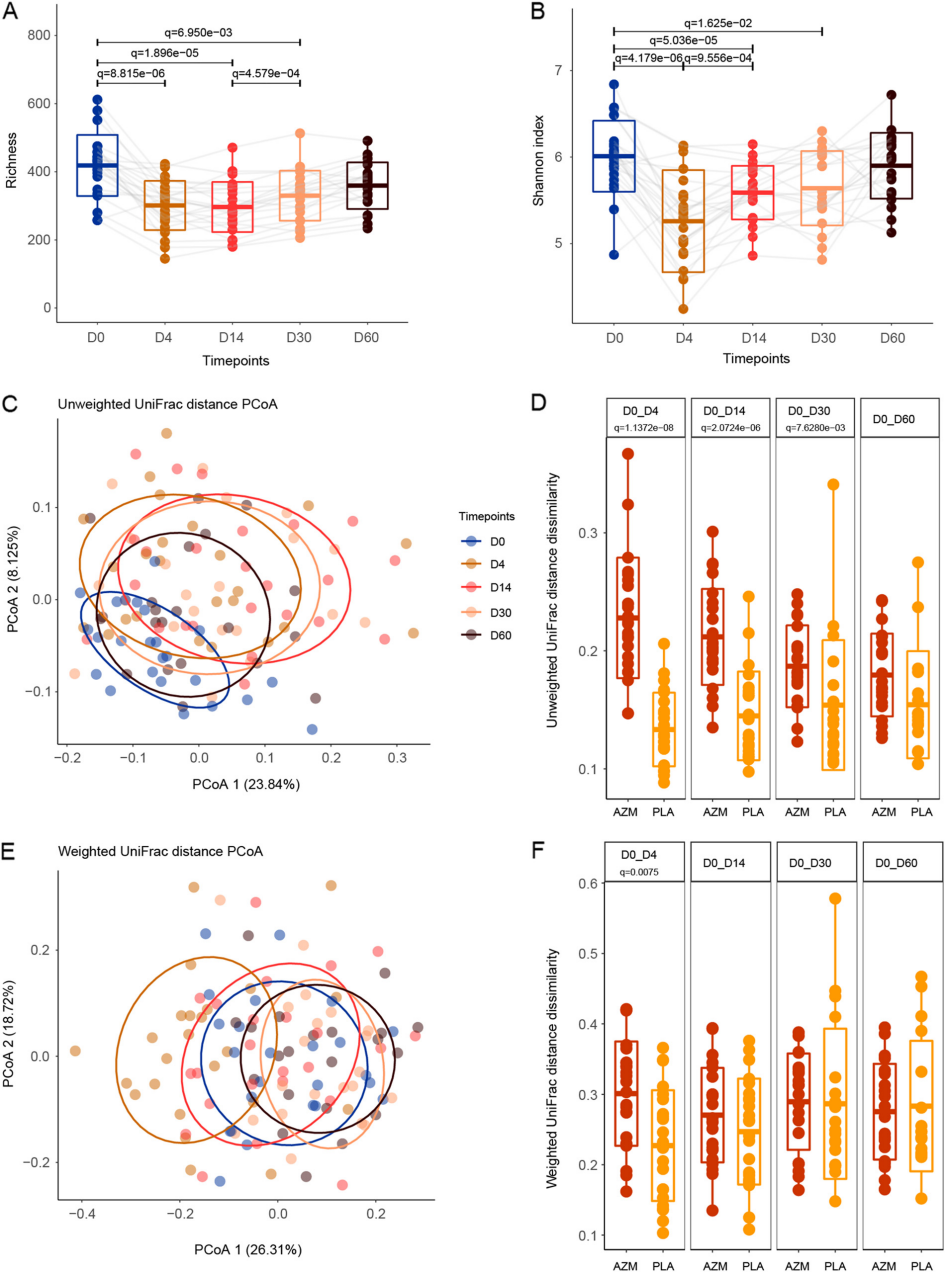
Airway microbial diversity and community composition changes in the azithromycin group. (A, B) The boxplots represent the diversity measures for the subjects (center line, mean; box limits, ±standard deviation; whisker limits, maximum/minimum). (A) Species richness. (B) Shannon index. The points are connected across time points by gray lines. (C, E) Principal coordinate analysis (PCoA) plots based on unweighted UniFrac distance (C) and weighted UniFrac distance (E). The ellipses represent the 68% confidence interval for each time point. (D) The boxplots in dark yellow showed that the extent of compositional changes between paired samples in the azithromycin (AZM) group during the study time (D0 versus D4, D0 versus D14, and D0 versus D30) were significantly different compared to the degree of variation over time observed for paired samples in the placebo (PLA) group (two-sided Wilcoxon rank sum test, *q* < 0.05), but the compositional changes between D0 and D60 did not differ from the placebo group. (F) The significant difference between paired samples in the azithromycin group was observed only at D4 compared to the shift for paired samples in the placebo group (two-sided Wilcoxon rank sum test, *q* = 0.0075), but the compositional changes between D0 and D14, between D0 and D30, and between D0 and D60 did not differ from the placebo group (center line, mean; box limits, ±standard deviation; whisker limits, maximum/minimum).

The baseline sputum microbiota of healthy volunteers (D0) showed no significant difference between the two groups (two-sided Student’s *t* test and PERMANOVA, *P* > 0.05 for all Table 1). In the azithromycin group, immediately after the azithromycin course was completed (D4), both the richness and the Shannon index were dramatically reduced compared to D0 (two-sided paired *t* test, *q* = 8.82 × 10^−6^ and *q* = 4.18 × 10^−6^, respectively; Fig. 3a and b). By D14, the species richness still remained decreased, but the Shannon index significantly increased compared to D4 (two-sided paired *t* test, *q* = 1, *q* = 9.56 × 10^−4^; Fig. 3a and b), suggesting that the relative abundance of surviving microorganisms affected by antibiotic started to recover. During the 2-month follow-up, the species richness significantly increased at D30 compared to D4 (two-sided paired *t* test, *q* = 0.019) and completely recovered at D60 compared to D0 (two-sided paired *t* test, *q* = 0.3; Fig. 3a). Similarly, the species richness of the oral cavity microbiota was dramatically reduced compared to D0 at D4 (two-sided paired *t* test, *q* = 7.08 × 10^−6^; Fig. S2a) after exposure to azithromycin, but it did not return to the baseline level by the end of observational period (*q* = 0.015; Fig. S2a).

In the placebo group, we found no significant differences in richness and Shannon index in the sputum microbiota of the healthy volunteers across the five time points (two-sided paired *t* test, all *q* > 0.05; Fig. S3a to b). This suggested that the α-diversity of airway microbiota remained stable through the study without antibiotic exposure.

In the azithromycin group, pairwise PERMANOVA testing using unweighted UniFrac distance showed significant compositional differences between time point D0 and time points D4, D14, D30, and D60 (*R*^2^ = 0.087, 0.108, 0.087, and 0.054; *q* = 0.01, 0.01, 0.01, and 0.05), principal coordinate analysis (PCoA) demonstrated that the sputum microbial compositions gradually returned toward their initial composition after profound differences at D4 (Fig. 3c). To verify whether the microbial profile shift after exposure to antibiotics was more obvious than the shift in the absence of antibiotics at the five time points, we also compared the unweighted UniFrac distance and weighted distance dissimilarities between two different time point sputum samples from the same subject. Compared to D0 sputum samples, in the azithromycin group, D4 showed the highest compositional differences, along with significantly descending magnitude over time. These differences were significantly larger than same-subject differences in the control participants (Fig. 3d and f). When we used weighted UniFrac distance accounting for relative abundance to perform pairwise PERMANOVA testing in the azithromycin group, the significant changes in microbial community composition compared to D0 were identified at D4 and D30, but not D14 and D60 (*R*^2^ = 0.104, 0.081, and 0.036, 0.044; *q* = 0.01, 0.02, 1, and 0.44; Fig. 3e). The oral microbiota community composition had similar variations after exposure to azithromycin (Fig. S2c and d). PERMANOVA testing using both unweighted UniFrac distance and weighted UniFrac distance found that the microbial community composition at D0 between the two niches was quite distinct (*R*^2^ = 0.04 and 0.05; *P* = 0.02 and 0.03; Fig. S2c and d). At D4, the microbiota between the two niches were similar (PERMANOVA testing using unweighted UniFrac distance and weighted UniFrac distance, *R*^2^ = 0.03 and 0.03; *q* = 0.11 and 0.13; Fig. S2c and d). However, by D14, D30, and D60, PERMANOVA testing showed that the difference of microbial composition between the oral cavity and the airway microbiota became obvious (weighted UniFrac distance, *R*^2^ = 0.04, 0.09, and 0.10; *q* = 0.044, 0.002, and 0.001; Fig. S2c and d). These results suggested that the oral cavity and the sputum microbiota of the same group volunteers showed a convergent trend after azithromycin disturbance at D4 but had different change patterns over time (Fig. S4; supplemental material).

### Sputum microbial taxonomic variation during 60-day follow-up.

**FIG 4:**
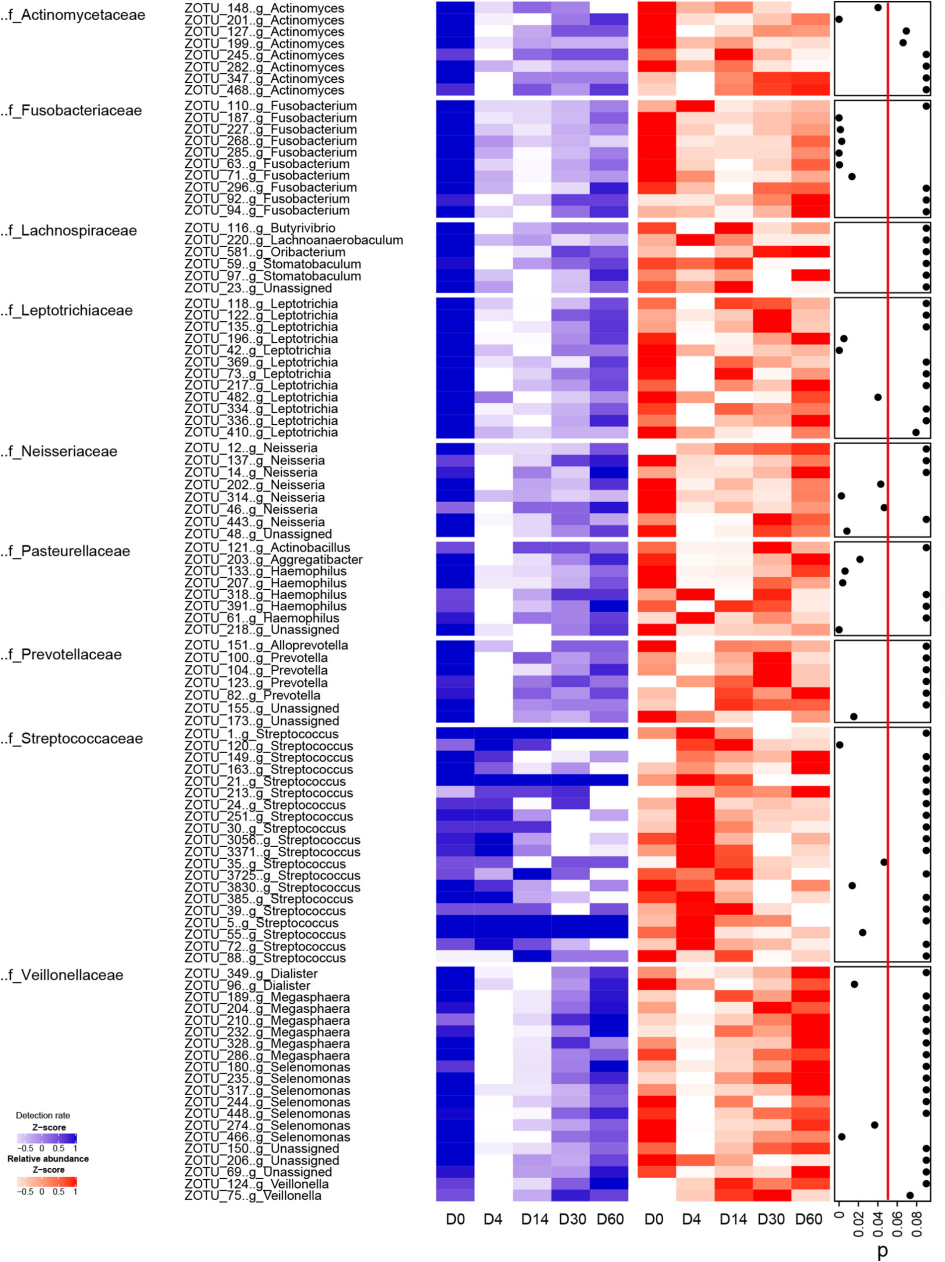
Microbial taxonomic variation after azithromycin administration during 60-day follow-up. The blue boxes mean the detection rate of zero-radius operational taxonomic units (ZOTUs) across the five time points. The detection rate describes the proportion of individual ZOTU appearing in the subjects. The red boxes mean the mean relative abundance of ZOTUs across the five time points. The relative abundance describes the mean percentage of the individual ZOTU in the whole subjects. *, *P* value ≥ 0.05: there is not a significant difference in the relative abundance of the species at D14 compared to D0; *P* value < 0.05: there is a significant difference in the relative abundance of the species at D14 compared to D0.

We investigated the sputum microbial taxonomic variation across the five time points by comparing the detection rate and relative abundance of the ZOTUs. We involved 330 ZOTUs for which the relative abundance was greater than 0.01% and the detection rate was greater than 50% at any time point in the analysis. After exposing to azithromycin, the detection rate of 114 ZOTUs decreased at D4. Twenty (17.54%) ZOTUs of them assigned to family *Veillonellaceae* (consisting of genera Selenomonas 6.14% and Megasphaera 5.26%), while 10.53% of the ZOTUs decreased at D4 belonged to genus Leptotrichia, and 8.72% to genus Fusobacterium, 7.02% to genus Actinomyces, 6.14% to genus Neisseria, and 4.39% to genus Haemophilus (Fig. 4; Table S2). About 84% ZOTUs for which the detection rate decreased at D4 returned to the D0 level at D60; others such as Fusobacterium ZOTUs, Haemophilus ZOTUs, Leptotrichia ZOTUs, and Actinomyces ZOTUs maintained a low detection rate and had not returned to the initial state by the end of observational period (Fig. 4; Table S2). During the 2-month follow-up, the detection rate of 150 ZOTUs remained stable; most of them belonged to the genus Prevotella (14%). The mean relative abundance of 64 ZOTUs decreased at D4, which was less than the ZOTU number in terms of detection rate. By D14, only 37 (32.46%) of the ZOTUs for which the detection rate decreased significantly between D0 and D4 exhibited significant differences in their relative abundance, while 74 (64.91%) of the above-mentioned ZOTUs showed differences in the detection rate, suggesting that the abundance of the species recovered fast once they reappeared in the microbiota community (Fig. 4; Table S2). We observed an enrichment of the genus Streptococcus ZOTUs in the relative abundance at D4; the difference of 40% of the Streptococcus ZOTUs was statistically significant. However, at D14, 80% of the Streptococcus ZOTUs rapidly decreased and returned to the baseline level (Fig. 4; Table S2).

The variations in the relative abundance and detection rate of oral microbiota were similar to those of sputum microbiota, but the unique changes were seen in the two niches (Fig. S4; supplemental material). We found no significant microbial variation in the detection rate and relative abundance in the placebo group across the five time points (Fig. S5).

### Attenuated resilience in response to outdoor environmental factor disturbance.

We found that the daily mean concentrations of outdoor PM_2.5_, PM_10_, air quality index (AQI) values, daily mean humidity, and temperature were significantly different (Kruskal-Wallis rank sum test, *P* < 0.05; Fig. S6) among the five periods. The multivariable PERMANOVA (Weighted UniFrac distance) was performed to explain outdoor environmental stresses on the sputum microbiota variation in the azithromycin group. By D4, the airway microbiota was affected only by antibiotic administration (*R*^2^ = 0.04, *P* = 0.024; humidity *R*^2^ = 0.01, *P* = 0.65; PM_2.5_ *R*^2^ = 0.03, *P* = 0.18; and PM_10_ *R*^2^ = 0.02, *P* = 0.28). By D30, the multivariable analysis showed that the high concentration of outdoor PM_2.5_ significantly contributed to the novel variability in microbiota composition (*R*^2^ = 0.084, *P* = 0.002), followed by PM_10_ (*R*^2^ = 0.073, *P* = 0.002), the level of humidity (*R*^2^ = 0.072, *P* = 0.003), and antibiotic exposure (*R*^2^ = 0.05, *P* = 0.013).

Dissimilar to the sputum microbiota, oral cavity microbiota can bear outdoor environmental stress and remain stable microbial community composition at D30 (PERMANOVA testing using weighted UniFrac distance, D0 versus D30, *R*^2^ = 0.03; *q* = 1; Fig. S2d) after azithromycin exposure. In the placebo group, outdoor environmental factors were not associated with the sputum microbiota in healthy volunteers (temperature *R*^2^ = 0.03, *P* = 0.21; humidity *R*^2^ = 0.02, *P* = 0.40; PM_2.5_ *R*^2^ = 0.02, *P* = 0.50; and PM_10_ *R*^2^ = 0.01, *P* = 0.72).

### Effects of azithromycin on airway microbial interactions within the microbial community network.

**FIG 5:**
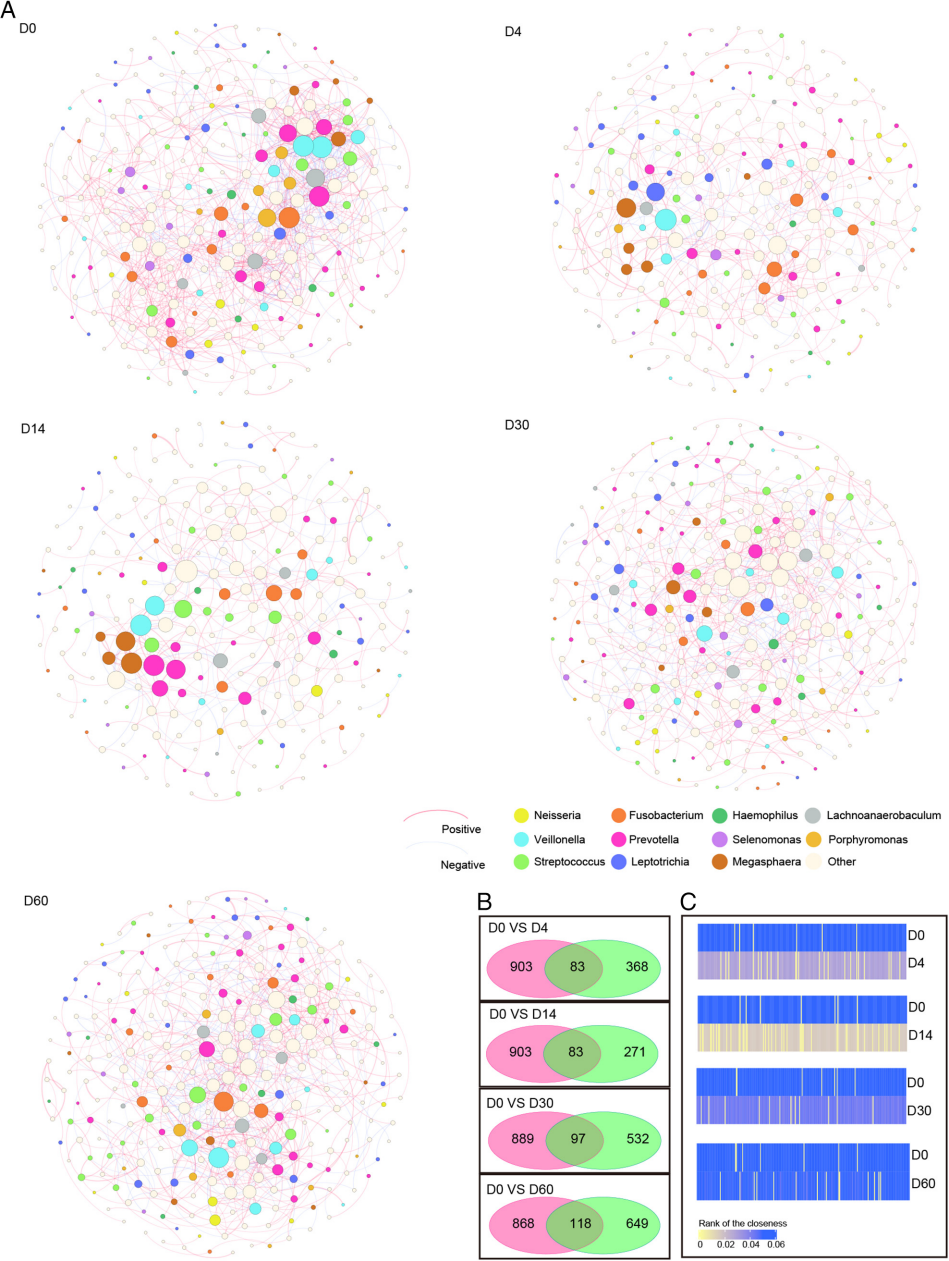
The network analysis in the azithromycin group. (A) Networks of co-occurring ZOTUs in airway microbiota for time points D0, D4, D14, D30, and D60. Nodes are colored by ZOTU genera, with size proportional to mean relative abundance and edge width proportional to confidence score. (B) The number of shared edges between network D0 and time points D4, D14, D30, and D60. (C) The closeness centralization of shared nodes between network D0 and time points D4, D14, D30, and D60.

To explore the changes of microbial interaction after exposure to azithromycin, a bacterial community network analysis was performed. The azithromycin group networks became less complicated and stabilized after the antibiotic exposure regarding decreases in the total number of vertices, edges, connectance, average degree, average clustering coefficient, and centralization closeness (Fig. S7). The co-occurrence patterns of microbial communities before and after azithromycin administration were quite distinct (Fig. 5a). We identified the importance of ZOTUs using closeness centralization scores and degree centralization scores. ZOTUs belonging to genera Veillonella and Fusobacterium set a central position at D0, and those assigned to genera Prevotella, Lachnoanaerobaculum, and Porphyromonas played important roles as well (Fig. 5a). After exposure to antibiotics, genus Veillonella still occupied the central position. Genus Leptotrichia came to the front, while the superiority of the genera Fusobacterium and Lachnoanaerobaculum in the network declined (Fig. 5a). By D60, genus Fusobacterium returned to its original position again (Fig. 5a). Along with the decrease in detection rate or relative abundance of the major species after administration of antibiotics, the synergistic effect in the co-occurrence networks occupied the main status (Fig. 5a). Despite having a few overlapped edges between time point D0 and other time point networks, the closeness of shared nodes was quite different. Although, by D14, the relative abundance of most ZOTUs showed no significant difference compared to D0, the closeness of them remained decreased (Fig. 5b and c). In the control group, genera Lachnoanaerobaculum, Leptotrichia, Prevotella, and Veillonella kept occupying the important positions within the framework along with time (Fig. S8a). The co-occurrence patterns of the microbial communities were quite similar, showing that they contained more overlapped edges, and the closeness of shared nodes exhibited very small differences among the five time point networks (Fig. S8b and c). Both positive and negative correlations existed in the networks, suggesting that the interactions among the species make the bacterial community stable when outdoor environment factors change. Bacterial community network analysis showed that the interactions among the species in the oral cavity microbiota community recovered earlier than sputum microbiota (Fig. S9 and S10). A detailed explanation can be seen in the supplementary information.

## DISCUSSION

The core findings of our study are that airway microbiota of healthy volunteers show resilience and abilities for recovery from azithromycin perturbation. There is a depletion of family *Veillonellaceae* and genera Leptotrichia, Fusobacterium, Actinomyces, Neisseria, and Haemophilus and an enrichment of genus Streptococcus immediately after azithromycin administration. The shifts in the diversity of the microbiology composition take 14 to 60 days to recover, depending on the measure used: UniFrac phylogenetic distance or α-diversity. Azithromycin exposure alters the microbial interactions within airway microbial community networks and decreases the ability of the airway microbiota resilience from outdoor environmental factors, especially PM_2.5_ stress. The influence of azithromycin on microbial interactions is noteworthy, although the relative abundance of most taxa has returned to the baseline level. Compared to the sputum microbiota, the oral cavity microbiota had similar variations in the relative abundance and detection rate of the taxa after exposure to azithromycin but a different pattern of change over time, which suggested that the induced sputum microbial data can represent the airway microbiota composition in healthy adults.

In our study, we found that exposure to azithromycin resulted in decreases in the detection rate and relative abundance of different genera belonging to *Veillonellaceae*, Leptotrichia, Neisseria, and Haemophilus. Those taxa may act as a major ecological change causing the decrease in α-diversity immediately after antibiotic administration. Although we treated the volunteers with a short-course antibiotic, the finding is similar to the previous studies. Long-term macrolide administration had a selective impact on H. influenzae or species assigned to *Pasteurellaceae* within airway microbiota in both asthma and non-cystic-fibrosis bronchiectasis patients (25, 26). Compared with placebo, 8-week azithromycin administration decreases 11 low abundance taxa, including Neisseria, and causes significantly reduced diversity in lung microbiota in emphysema patients (27).

In contrast, the relative abundance of taxa belonging to Streptococcus increases immediately after azithromycin administration. Among asthma patients, the azithromycin administration is correlated with an increase in cultured azithromycin-resistant Viridans streptococci, in line with the increased abundance of Streptococcus reported here (25). The macrolide resistance in Streptococcus spp. is increasing around the world in recent years (28–30). In China, more than 90% of the S. pneumoniae isolates are resistant to macrolide in both children and adults (31, 32). A previous study showed that in the oral streptococcal flora of healthy volunteers, the proportion of macrolide-resistant streptococci was up to 30%, 85% of the isolates carried the macrolide-resistance gene *mef*, and 18% the *erm(B)* gene (33). Burr et al. (34) showed that proportional macrolide resistance in human oropharyngeal streptococci increased with azithromycin exposure, remaining above baseline levels for the azithromycin group at washout. High antibiotic resistance may provide those species with an advantage to effectively colonize the airway after administration. Fortunately, the upgrades of Streptococcus species are transient and return to the D0 level after antibiotic ceases in our study.

PM_2.5_ exposure is associated with increased all-cause and respiratory mortality and exacerbations of chronic lung diseases (35, 36). Outdoor PM_2.5_ exposure was found to alter human pharyngeal and airway microbiota composition because atmospheric particulate matter commonly carried a variety of microbes (16, 17, 37). During our study period, the outdoor concentration of PM_2.5_ was very high in the week around time point D30. However, we did not observe variations in the airway microbiota of placebo group subjects. The reason may be that the microbiota communities can show remarkable stability against perturbation, and the respiratory microbiota normally has the ability to deal with the inhaled pollutants (7, 38). The microbiota stability may comprise two concepts. One is resistance, meaning that a given microbiota stands unchanged in response to a disturbance, and the other is resilience, which describes the microbiota’s ability to return to the predisturbed state (39). On the contrary, the airway microbiota of azithromycin group subjects, despite an almost re-established microbial ecology after exposure to antibiotics, cannot bear the same burden of PM_2.5_ perturbation and has a novel shift in microbial community composition at D30. From the previous studies, we find that the individuals with dysregulated respiratory tract conditions, such as patients with respiratory illness and smokers, are particularly susceptible to environmental stress (40). Therefore, in our study, the stability of airway microbiota may have decreased because of azithromycin exposure, resulting in attenuated resilience in response to outdoor environmental factors.

We performed network analysis to support the above-mentioned assumption. The functional stability of microbial communities depends on the complicated microbe-microbe interactions (41). Studies on soil microbiota confirm that the network complexity is consistent with the ability to cope with diverse environmental changes (42). After exposure to azithromycin, the airway microbiota networks in our study became simpler, which may account for the attenuated resilience. The combination of both mutualistic and antagonistic correlations embraced in the networks is important to keep ecological equilibrium (43). The microorganisms within the communities can cooperate and compete for nutrition to prevent the growth or decline of certain one (38). In our results, the weakening antagonistic effects due to losing some species contribute to the novel variation in airway microbiota when outdoor environment factors change. It is reported that the mode of action of keystone taxa that mediates multiple interactions within networks is associated with the stability of the microbial communities (44). Our study finds that antibiotic perturbation targets the crucial taxa by altering the position of them in the framework, which may, in turn, precipitate the microbiota structure fragility. The influence of azithromycin on microbial interactions within airway microbiota is noteworthy, although the relative abundance of most taxa returns to the baseline level. It is expected that antibiotic exposure and high levels of outdoor air pollution will lead to a fragile microbial community more prone to disease than a high PM_2.5_ only. Unfortunately, we cannot prove the assumption because of the limited sample size and the complexity of the relationship between airway microbiota and the development of respiratory tract infections.

Our study has following limitations. First, we use induced sputum to investigate the variation in airway microbiota after antibiotic intervention. The induced sputum is a variable mixture that may contain materials from human airways and oral cavities. We asked the subjects to fast for 4 to 6 h and clean their oral cavity. To determine the contribution of each source, oral washes were also performed by having the subjects gargle with 10 mL sterile 0.9% saline for 60 s immediately before sputum induction. We found that the bacterial DNA load in oral wash samples is significantly lower than sputum samples (Fig. S11). Although the oral wash samples are commonly used in oral cavity microbiota studies, the bacterial load being lower in the oral wash may be the result of sample dilution. The oral cavity microbiota has higher species richness and a significantly different microbial community composition compared to sputum microbiota. Compared to the sputum microbiota, the oral cavity microbiota has a different change pattern and recovers earlier after antibiotic exposure (Fig. S12 to S16). However, for successful 16S rRNA gene library preparation, we increased the volume of oral wash samples up to 3 mL for DNA extraction. This is not consistent with the volume of the induced sputum (750 μL), which may decrease the comparability of the sequencing data.

Second, the human airway microbiome may be influenced by numerous factors. For example, there was a lack of some data on diet and its possible effects on the airway or oral microbiota. To avoid deviations as much as possible, we strictly executed a randomized, double-blind, placebo-controlled pattern across the entire study and experimentation process. The stability in airway microbiota of the placebo group subjects gave us evidence to believe our conclusions drawn from the azithromycin group. Third, we did not explore the relationship between the antibiotic resistance and airway microbiota. It is important to figure what or how the streptococci resist the drug effect and how they lose it following re-establishment of the microbial structure, but the study design of this paper could not answer that. In a future study, we will focus on this important question. At last, we followed up with the volunteers for only 60 days; the longer-term effects after azithromycin administration demand more attention in future.

### Conclusions.

In summary, we conclude that azithromycin exposure has transient effects on the airway microbial community composition of healthy adults. The induced sputum microbial data can represent the airway microbiota composition in healthy adults. Azithromycin may alter the microbial interactions within airway microbial community networks and decrease the airway microbiota resilience from outdoor environmental perturbations, especially PM_2.5_ stress. The influence of azithromycin on microbial interactions was noteworthy, although the relative abundance of most taxa returned to the baseline level 30 days after exposure.

## MATERIALS AND METHODS

### Ethics statement.

The study was reviewed and approved by the China-Japan Friendship Hospital Ethics Committee (approval No. 2018-120-K86). All subjects provided written consent.

### Registration.

This trial was riled with the Chinese Clinical Trial Registry (ChiCTR1800018494) in September 2018 (http://www.chictr.org.cn/edit.aspx?pid=31269&htm=4).

### Study design and population.

This study was a randomized, double-blind, placebo-controlled trial to investigate the change of airway microbiota after azithromycin exposure among healthy adults. We distributed research posters in communities and recruited all the healthy participants from the community setting. Inclusion criteria for the healthy volunteers were men and women aged over 18 years who had no history of smoking, no history of any underlying diseases, and no existing abnormal symptoms. Detailed inclusion and exclusion criteria are provided in supplementary information (Table S4). Based on the selection criteria, we recruited 48 healthy volunteers randomly assigned to either the azithromycin or the placebo group (1:1) using a computer-generated random number table. We administered 500 mg azithromycin or 500 mg starch tablets once daily for 3 days. The drugs were contained in an identical opaque white capsule and packed in sealed kraft paper bags. All healthy volunteers, investigators, and study research staff were masked to intervention allocation.

### Sputum collection.

The healthy volunteers were asked to fast for 4 to 6 h and clean their oral cavity, including brushing teeth and gargling with 10 mL 0.9% saline for 60 s at our laboratory immediately before the sputum induction. The oral wash samples were collected. The sterile saline was used as an oral wash control. Then, they were subjected to inhalation of 4.5% sterile hypertonic saline nebulized from an inhalation delivery (PARI, Germany) for 10 min and endeavored to cough throughout the process. Most subjects could expectorate an adequate sample (sputum of ≥1 mL with as little saliva as possible) after the first inhalation. For those who failed to meet the requirements for sputum, we extended the duration of aerosol inhalation cumulatively up to 60 min with a pause every 10 min and allowed them to cough for the second time later. Sputum was collected in sterile containers and weighed, and 2 mL of 0.1% DTT (Sigma-Aldrich, Poole, UK) in phosphate-buffered saline (PBS) was added for liquefaction. The 0.1% DTT-PBS in sputum collection cups and sterile saline in the atomization device served as negative-control samples. After incubation at room temperature for 15 min on a rolling mixer, all the samples were stored in sterile tubes at −80°C immediately until processing. The sputum samples were collected at the day before drug administration (D0), the day after the treatment course was completed (D4), and 14, 30, and 60 days postdosing. We asked the participants to maintain a consistent time for each sample collection day.

### Outdoor environmental and volunteers’ follow-up information collection.

We collected outdoor environmental information that might influence the airway microbiota, including air pollution data, humidity, temperature, and history of leaving Beijing city during the study period. We obtained the daily mean concentration of outdoor PM_2.5_ and PM_10_, air quality index (AQI) values, daily mean humidity, and temperature in Beijing city from an online platform monitoring air quality in China (https://www.aqistudy.cn/historydata/). Relevant information recorded before every sampling included whether the subjects developed respiratory tract infection symptoms and history of antibiotic administration.

### DNA extraction and 16S rRNA gene sequencing.

DNA extraction from sputum and oral wash samples, paralleled with experimental control samples, was performed using the Maxwell RSC whole blood DNA kit (Promega, USA). A sample of 750 μL was transferred to the Eppendorf tube and centrifuged for 5 min at 14,000 × *g*. The supernatant was discarded, and precipitation was resuspended in PBS to 720 μL with 80 μL proteinase K (Tiangen, China) added in. Then, we transferred resuspended material to a lysing matrix A tube (Qbiogene, Carlsbad, CA). The cells were lysed by bead beating using a FastPrep system (Qbiogene, Carlsbad, CA) for 3 cycles of 30 s at 6.0 m/s, and the mixture was subsequently incubated at 56°C for 1 h. After centrifugation for 5 min at 14,000 rpm, 500 μL supernatant was then transferred to the first well of the cartridge. A total of 70 μL of elution buffer was added to the bottom of each elution tube. Extracted DNA was quantified using a Qubit 3.0 fluorometer (Thermo Fisher Scientific, Waltham, MA, USA) and stored at −20°C.

The V3-V4 hypervariable region of the 16S rRNA gene of all samples was amplified by PCR according to the 16S Metagenomic Sequencing Library Preparation guide provided by Illumina (45). The amplicon primers were as follows: forward primer, 5′-TCGTCGGCAGCGTCAGATGTGTATAAGAGACAGCCTACGGGNGGCWGCAG; and reverse primer, 5′-GTCTCGTGGGCTCGGAGATGTGTATAAGAGACAGGACTACHVGGGTATCTAATCC. The PCR conditions were 95°C for 3 min; followed by 25 cycles of 95°C for 30 s, 56°C for 30 s, and 72°C for 30 s; and then 72°C for 5 min. The amplicons were performed in 25-μL reactions with 12.5 ng template DNA. Index PCR was the second stage PCR step that attached dual indices and Illumina sequencing adapters using the Nextera XT index kit, index primer 1 (N7xx), and Index Primer 2 (S5xx) (Illumina, San Diego, CA) in a 50-μL reaction. The PCR conditions were 95°C for 3 min; 8 cycles at 95°C for 30 s, 55°C for 30 s, and 72°C for 30 s; and then 72°C for 5 min. The resulting amplicons were purified using Agencourt AMPure XP (Beckman Coulter) and quantified using a Qubit 3.0 fluorometer. The pooled library, including negative controls, was sequenced on an Illumina Miseq platform (Illumina, San Diego, CA, USA) using pair-end sequencing (2 × 300 bp).

### Bacterial DNA quantification.

Bacterial DNA of sputum samples was quantified using a QX200 droplet digital PCR system (Bio-Rad, Hercules, CA, USA). Two replicates were used per sample. Primers, cycling conditions, and workflow were performed according to a previously published protocol (3).

### Statistical analysis.

The sequencing data were processed and analyzed using the software VSEARCH version 2.7.1 (46), USEARCH version 10.0 (47). The sequencing data were merged (minimum 50-bp overlap), trimmed of primers and indexes, and quality filtered (fastq maxee rate 0.01) with VSEARCH version 2.7.1. The reads were denoised into zero-radius operational taxonomic units (ZOTUs) with UNOISE3 (48), and the chimeras were filtered using the SILVA version 123 reference database. Taxonomic assignment of ZOTUs was performed by SINTAX (49).

Statistical analysis was performed in R version 3.6.1 via the Rstudio interface. To identify the contaminating DNA coming from experiment operation, the decontam package was used. The feature table covering all samples and the concentration of DNA in each eligible library as measured by fluorescent intensity were input to the function. The species richness, Shannon index, unweighted UniFrac distance, and weighted UniFrac distance were computed, and ordination analyses were conducted using the vegan, ape, ggplot2, and GUniFrac packages with all ZOTUs. Principal coordinates analysis and permutational analysis of variance (PERMANOVA) was performed based on both unweighted UniFrac distance and weighted UniFrac distance. Multivariable PERMANOVA was performed using weighted UniFrac distance. The diversity comparations between the same group were calculated by paired *t* test or Wilcoxon signed-rank test. The diversity comparations between the different groups were calculated by Student’s *t* test or Wilcoxon rank sum test. We performed linear mixed model and a McNemar-Bowker test to compare the variation in the relative abundance and detection rate of the ZOTUs whose relative abundance was greater than 0.01% and detection rate was greater than 50% at any time point, respectively. Network analysis was used to explore the co-occurrence patterns of ZOTUs within the groups. We involved the ZOTUs for which the relative abundance was greater than 0.01% and the detection rate was greater than 50% at any time point to obtain Spearman rank correlation matrix (*r* ≥ 0.6 and *q* ≤ 0.05). The networks were constructed and visualized using the igraph package and the gephi program. *P* values were adjusted using the Bonferroni-control procedure. The heat maps were visualized by ComplexHeatmap package (50).

### Data availability.

The sequence data have been deposited in the NCBI Sequence Read Archive under accession PRJNA565553. Feature, taxonomy, metadata tables, and a reproducible workflow of the analysis are available for download at https://github.com/Zoey-Du/Sputum_Microbiota.
