# Supplementary material for: Azithromycin Exposure Induces Transient Microbial Composition Shifts and Decreases the Airway Microbiota Resilience from Outdoor PM2.5 Stress in Healthy Adults: a Randomized, Double-Blind, Placebo-Controlled Trial
Source: Microbiol Spectr. 2023 Apr 24;11(3):e02066-22. doi: 10.1128/spectrum.02066-22 (PMC10269807; doi:10.1128/spectrum.02066-22)
Supplement: Supplemental file 1 — Supplemental material. Download spectrum.02066-22-s0001.docx, DOCX file, 8.9 MB [file spectrum.02066-22-s0001.docx]

The induced sputum is a variable mixture that may contain materials from human airways and oral cavity. We ask the subjects to fast for 4-6 hours and clean oral cavity to decrease the biomass of the oral samples. To determine the contribution of each source, oral washes are also performed by having the subjects gargle with 10 ml sterile 0.9% saline for 60 seconds immediately before sputum induction. We compared induced sputum and oral wash bacteria using 16S rRNA gene quantification and sequencing.

**Low bacterial biomass of oral wash samples**

We quantified the bacterial DNA of every oral wash sample in azithromycin group using droplet digital PCR system. The bacterial DNA load in oral wash samples (the first quartile and the third quartile, 1.1 x 10^6^~4.5 x 10^6^ copies/g) was significantly lower than sputum samples across different timepoints (Wilcoxon signed-rank test, q<0.05 for all Fig. S11).

**Contamination identification on oral wash samples sequencing in azithromycin group**

For successful 16S rRNA gene library preparation, we increased the volume of oral wash samples, up to 3 ml, for DNA extraction. The methods of DNA extraction, 16S rRNA gene sequencing and statistical analysis workflow were consistent with those of sputum samples. Three samples were removed because of insufficient sequencing for microbial analysis. The microbial community composition between oral wash samples and control samples was quite distinct based on both unweighted UniFrac and weighted UniFrac distance (PERMANOVA, R^2^=0.09, 0.45; P=0.001,0.001 Fig. S12a-b). The five Zero-radius Operational Taxonomic Units (ZOTUs, 100% sequence similarity) detected in oral wash samples with the highest abundance comprised 37.51% of all sequences, while comprising 1.16% of all sequences detected in control samples (Fig. S12c). The most abundant ZOTU detected in control specimens (ZOTU4) accounted for only 0.45% of all sequences detected in oral wash specimens (Fig. S12d). We performed microbial ecology analysis using the decontam package whose function was to identify contaminants in sequencing data based on statistical approach. We used both the frequency and prevalence methods with threshold 0.1 and 0.5 respectively and confirmed 13 contaminants ZOTUs (ZOTU107, ZOTU1087, ZOTU139, ZOTU1444, ZOTU2117, ZOTU2169, ZOTU2582, ZOTU2588, ZOTU2809, ZOTU3154, ZOTU32, ZOTU3333, ZOTU3417) in our data (Fig. S12e-f, Table S1). The relative abundance of the 13 ZOTUs were very low in both oral wash and negative control samples (comprising 2.29 x 10^-2^%, 3.14 x 10^-4^%, 1.24 x 10^-2^%, 2.98 x 10^-4^%, 5.78 x 10^-6^%, 9.63 x 10^-6^%, 2.27 x 10^-4^%, 2.06 x 10^-4^%, 7.7 x 10^-6^%, 7.7 x 10^-5^%, 8.44 x 10^-2^%, 4.81 x 10^-5^%, 6.93 x 10^-5^% of all oral wash samples sequences and 3.0%, 3.71 x 10^-2^%, 1.99%, 5.3 x 10^-2^%, 5.2 x 10^-3^%, 5.15 x 10^-3^%, 4.14 x 10^-5^%, 2.07 x 10^-5^%, 6.83 x 10^-4^%, 2.07 x 10^-5^%, 12.42%, 7.66 x 10^-4^%, 2.07 x 10^-5^% of all negative control samples sequences).

**Similar but not identical microbial profile between the oral cavity and the airway microbiota**

In azithromycin group, the species richness of baseline oral cavity microbiota of healthy volunteers (D0) was significantly higher than the sputum microbiota (488.92 ± 90.02 vs 418.62 ± 89.73 two-sided paired t-test, q=0.0003 Fig. S2a-b). PERMANOVA testing using both unweighted UniFrac distance and weighted UniFrac distance found that the microbial community composition at D0 between the two niches was quite distinct (R^2^=0.04, 0.05; P=0.02, 0.03 Fig. S2c-d). As can be seen in the heatmap of detection rate, about 4.2% of ZOTUs (whose relative abundance was greater than 0.01% and detection rate was greater than 50% at any timepoint) in the sputum microbiota didn’t appear in the oral wash microbiota (Fig. S4a). Conversely, about 21.39% of ZOTUs were unique in the oral cavity microbiota (Fig. S4a). Although the clustering of taxa with high relative abundance were similar between the oral cavity and airway at ZOTU level (Fig. S4b), the relative abundance of families *Prevotellaceae*, *Veillonellaceae*, *Porphyromonadaceae*, *Micrococcaceae*, *Peptostreptococcaceae, Bacillales_Incertae_Sedis_XI* and genera *Prevotella*, *Porphyromonas*, *Veillonella*, *Rothia*, *Gemella* were significantly higher in the sputum microbiota while families *Flavobacteriaceae*, *Pasteurellaceae*, *Fusobacteriaceae, Burkholderiaceae, Carnobacteriaceae* and genera *Capnocytophaga*, *Fusobacterium*, *Haemophilus*, *Granulicatella* were higher in the oral wash microbiota (Wilcoxon signed-rank test, q<0.05 for all Fig. S4c-d). No significant microbial variation in biodiversity, the detection rate and relative abundance were found in the placebo group across the five timepoints in oral wash microbiota (Fig. S13).

**Different process of variation in oral cavity microbial diversity after antibiotic exposure**

After exposure to azithromycin, the species richness of the oral cavity microbiota was dramatically reduced compared to D0 at D4 (two-sided paired t-test, q=7.08x10^-6^ Fig. S2a) and didn’t return to the baseline level by the end of observational period (q=0.01 Fig. S2a). Similarly, pairwise PERMANOVA testing using unweighted UniFrac distance showed significant compositional differences between timepoint D0 and timepoints D4, D14, D30 and D60 (R^2^=0.10, 0.14, 0.13, 0.10; q=0.01, 0.01, 0.01, 0.01 Fig. S2c). The Shannon index significantly decreased at D4 compared to D0 (two-sided paired t-test, q=1.68x10^-6^ Fig. S2b), unlike the shifts in the sputum microbiota, and came back to the D0 level at D14 (q=1 Fig. S2b). When we used weighted UniFrac distance perform pairwise PERMANOVA testing, the significant change in microbial community composition compared to D0 was only identified at D4 (R^2^=0.085, P=0.01 Fig. S2d).

By the timepoints D4, D14, D30 and D60, there weren’t significant difference in species richness and Shannon index between the oral cavity and the airway microbiota (two-sided paired t-test, q>0.05 Fig. S2a-b). The microbial community composition between the two niches were similar at D4 (PERMANOVA testing using unweighted UniFrac distance and weighted UniFrac distance, R^2^=0.03, 0.03; P=0.11, 0.13 Fig. S2c-d). However, by D14, D30 and D60, PERMANOVA testing showed that the difference of microbial composition between the oral cavity and the airway microbiota became obvious (weighted UniFrac distance, R^2^=0.04, 0.09, 0.10; P=0.044, 0.002, 0.001 Fig. S2c-d). These results suggested that the oral cavity and the sputum microbiota of the same group volunteers showed a convergent trend after azithromycin disturbance at D4 while had different change patterns over time.

We found no significant differences in alpha and beta diversity among the healthy volunteers in the placebo group across the five timepoints (Fig. S14).

**Microbial taxonomic variation during 60 days’ follow-up**

We involved 388 ZOTUs of the oral wash microbiota and 330 ZOTUs of the sputum microbiota whose relative abundance was greater than 0.01% and detection rate was greater than 50% at any timepoint to analyze. After exposing to azithromycin, in the oral cavity microbiota, the detection rate of 131 ZOTUs decreased at D4, including some species, such as family *Veillonellaceae* (15.27% consisting of genera *Selenomonas* 7.63% and *Megasphaera* 3.05%), genus *Leptotrichia* (15.27%), genus *Fusobacterium* (10.69%), genus *Actinomyces* (8.40%), genus *Haemophilus* (4.58%), genus *Neisseria* (3.05%), which were similar to the variation in the sputum microbiota (Fig. S15, Table S2). Most of the ZOTUs returned to the D0 level at D14 but 23.66% of the above mentioned ZOTUs still remained low detection rate at D60, which was higher than the ratio (15.79%) in the sputum microbiota (Fig. S16a, Table S3). Among 388 ZOTUs of the oral microbiota, the detection rate of 257 ZOTUs returned to the D0 level at D4 and 14.01% of them exhibited significant differences compared to D0 again at D14 (Fig. S16a, Table S3). The relative abundance of 302 ZOTUs were not significantly different between D0 and D4 and 14.9% of those ZOTUs had novel variation in the relative abundance at D14 (Fig. S16b, Table S3). Among 330 ZOTUs of the sputum microbiota, the detection rate of 216 ZOTUs returned to the D0 level at D4 and only 9.7% of them exhibited significant differences compared to D0 again at D14 (Fig. 5a, Table S3). The relative abundance of 258 ZOTUs were not significantly different between D0 and D4 while 14.73% of those ZOTUs had novel variation in the relative abundance at D14 (Fig. 5b, Table S3).

By D4, 35 ZOTUs (10.61%) had variation in the detection rate in the airway microbiota while remained stable in the oral cavity microbiota. By timepoints D14, D30 and D60, the ratio was 8.78% (29), 7.27% (24) and 43.33% (4.55) respectively (Fig. S16c). The unique changes in the relative abundance were seen in 31 ZOTUs (9.39%), 34 ZOTUs (10.3%), 33 ZOTUs (10%) and 22 ZOTUs (6.67%) of the sputum microbiota at D4, D14, D30 and D60 respectively (Fig. S16d). We included the ZOTUs whose detection rate or relative abundance had shifts only in the sputum microbiota across different timepoints for PERMANOVA testing. The results showed that the individual changes of the ZOTUs owned by the sputum microbiota had the ability to cause the variation in the airway microbiota composition after exposure to azithromycin (Table S5).

Table S5. PERMANOVA testing with unique ZOTUs

|  | The number of unique ZOTUs in detection rate variation | The number of unique ZOTUs in relative abundance variation | Total number of ZOTUs of both | Based on unweighted UniFrac distance | |  | Based on weighted UniFrac distance | |
| --- | --- | --- | --- | --- | --- | --- | --- | --- |
|  |  |  |  | R^2^ | P |  | R^2^ | P |
| D0_D4 | 35 | 31 | 58 | 0.101 | 0.001 |  | 0.049 | 0.04 |
| D0_D14 | 29 | 34 | 58 | 0.122 | 0.001 |  | 0.046 | 0.051 |
| D0_D30 | 24 | 33 | 53 | 0.121 | 0.001 |  | 0.090 | 0.001 |
| D0_D60 | 15 | 22 | 35 | 0.104 | 0.001 |  | 0.057 | 0.019 |

**Dissimilar effects of azithromycin on the oral cavity microbiota network**

In azithromycin group, by D4, there was significant change in microbial community composition compared to D0 in oral cavity (R^2^=0.085, P=0.01). However, multivariable analysis didn’t find the independent relation between azithromycin administration and microbiota shifts (Weighted UniFrac distance, R^2^=0.03; q=0.17). It seemed that more factors could affect the oral microbiota biodiversity. Besides, dissimilar to the sputum microbiota, oral cavity microbiota can bear the high burden of PM_2.5_ pollution and remain stable microbial community composition at D30 (PERMANOVA testing using weighted UniFrac distance, R^2^=0.18; q=1 Fig. S2d), suggesting that the oral cavity microbial interactions within the microbial community might be different from those of the sputum microbiota after azithromycin exposure. We performed bacterial community network analysis on the oral cavity microbiota. After azithromycin administration, the oral cavity microbiota structure network became less complicated as well. Compared to the sputum microbiota structure network, the topological parameters of the oral cavity microbiota began to increase at D14 (Fig. S9). ZOTUs belonging to genera *Streptococcus*, *Lachnoanaerobaculum,* *Prevotella* and *Veillonella* played the important roles at D0. After exposure to antibiotics, genera *Veillonella* still set a central position while genera *Streptococcus, Lachnoanaerobaculum, Prevotella* superiority in the network declined. By D14, genera *Streptococcus, Lachnoanaerobaculum, Prevotella* had returned to their original position (Fig. S10a). The number of overlapped edges were large and the closeness of shared nodes was similar between the oral microbiota network and the sputum microbiota network at timepoint D0 (Fig. S10b). Despite having a few overlapped edges between the two niches’ networks at timepoint D4 or D14, the closeness of shared nodes was quite different. By D14, the closeness of the ZOTUs in the oral wash microbiota was higher than those in sputum microbiota, suggesting that the interactions among the species recovered earlier than the sputum microbiota (Fig. S10b-c).

**Table S4.** The inclusion and exclusion criteria for the subjects

| **Inclusion criteria** |
| --- |
| - Men and women aged ≥ 18 years. |
| - No history of smoking. |
| - No history of any pulmonary disease (e.g. asthma or chronic obstructive pulmonary disease) or cardiac disease (e.g. arrhythmia or congestive heart failure) or hepatic diseases (e.g. hepatitis) or renal disease (e.g. nephrotic syndrome) |
| - No history of abnormal pulmonary function testing |
| **Exclusion criteria** |
| - Exposure to antibiotics or immunosuppressive medications in the past 3 months |
| - Any medication allergies |
| - History of an acute respiratory infection deﬁned as fever, cough, or upper respiratory symptoms in the previous 4 weeks |
| - Abnormal results of any of the screening tests, including blood routine, liver function, electrocardiograph and urine pregnancy test |

**Figure legends**

Fig. S1. Procedural contaminations on sputum samples sequencing analysis. (A) PCoA plot based on unweighted UniFrac distance (A) and weighted UniFrac distance (B) between sputum samples and negative control samples. The ellipses represent the 68% confidence interval for each timepoint. ZOTUs are ranked in descending order of mean relative abundance of sputum samples (C) and control samples (D). Decontam package confirms 3 contaminants ZOTUs (ZOTU1864, ZOTU2014, ZOTU2236) in our data using both the frequency (E) and prevalence (F) methods with threshold 0.1 and 0.5 respectively. * a noncontaminant ZOTU.

Fig. S2. Oral cavity and airway microbial diversity and community composition in azithromycin group across the five timepoints. (A) Species richness. (B) Shannon index. (C) PCoA plot based on unweighted UniFrac distance for oral cavity microbiota. (D) PCoA plot based on weighted UniFrac distance for oral cavity microbiota. The ellipses represent the 68% confidence interval for each timepoint.

Fig. S3. Airway microbial diversity and community composition in placebo group. (A) Species richness. (B) Shannon index. The boxplots represent the diversity measures for the subjects (centre line, mean; box limits, ±standard deviation; whisker limits, maximum/minimum). The points are connected across timepoints by grey lines. (C) PCoA plot based on unweighted UniFrac distance. (D) PCoA plot based on weighted UniFrac distance. The ellipses represent the 68% confidence interval for each timepoint.

Fig. S4. The microbial profile of oral wash samples and sputum samples in azithromycin group. (A) Clustering of the oral wash sample microbiota at ZOTU level according to the detection rate. (B) Clustering of the oral wash sample microbiota at ZOTU level according to the mean relative abundance. Average relative taxa abundance comparisons between oral cavity and sputum microbiota at the family (C) and genus (D) levels.

Fig. S5. Microbial taxonomic variation in placebo group in airway during 60 days’ follow-up.

Fig. S6. The daily mean outdoor air pollution data and temperature and humidity during the sputum samples collecting. * AQI [161(73)ug/m^3^], the concentration of outdoor PM_2.5_ [90(36)ug/m^3^] and PM_10_ [213(159)ug/m^3^] are highest at D30 among the five timepoints (Nemenyi test, q<0.05).

Fig. S7. The topological properties of microbial networks in azithromycin and placebo groups.

Fig. S8. The network analysis in placebo group. (A) Networks of co-occurring ZOTUs in airway microbiota for timepoints D0, D4, D14, D30 and D60. Nodes are colored by ZOTU genera, with size proportional to mean relative abundance, and edge width proportional to confidence score. (B) The number of shared edges between network D0 and timepoints D4, D14, D30, D60. (C) The closeness centralization of shared nodes between network D0 and timepoints D4, D14, D30, D60.

Fig. S9. The topological properties of microbial networks in oral cavity and airway microbiota.

Fig. S10. The network analysis in oral cavity microbiota in azithromycin group. (A) Networks of co-occurring ZOTUs in oral cavity microbiota for timepoints D0, D4, D14, D30 and D60. Nodes are colored by ZOTU genera, with size proportional to mean relative abundance, and edge width proportional to confidence score. (B) The number of shared edges between oral cavity and airway networks at timepoints D0, D4, D14, D30, D60. (C) The closeness centralization of shared nodes between oral cavity and airway networks at timepoints D0, D4, D14, D30, D60.

Fig. S11. The bacterial DNA burden in oral wash samples and sputum samples.

Fig. S12. Procedural contaminations on oral wash samples sequencing analysis. (A) PCoA plot based on unweighted UniFrac distance (A) and weighted UniFrac distance (B) between oral wash samples and negative control samples. The ellipses represent the 68% confidence interval for each timepoint. ZOTUs are ranked in descending order of mean relative abundance of oral wash samples (C) and control samples (D). Decontam package confirms 7 contaminants ZOTUs in our data using both the frequency (E) and prevalence (F) methods with threshold 0.1 and 0.5 respectively. * a noncontaminant ZOTU.

Fig. S13. The microbial profile of oral wash samples and sputum samples in placebo group. (A) Clustering of the oral wash sample microbiota at ZOTU level according to the detection rate. (B) Clustering of the oral wash sample microbiota at ZOTU level according to the mean relative abundance. Average relative taxa abundance comparisons between oral cavity and sputum microbiota at the family (C) and genus (D) levels.

Fig. S14. Oral cavity and airway microbial diversity and community composition in placebo group across the five timepoints. (A) Species richness. (B) Shannon index. (C) PCoA plot based on unweighted UniFrac distance for oral cavity microbiota. (D) PCoA plot based on weighted UniFrac distance for oral cavity microbiota. The ellipses represent the 68% confidence interval for each timepoint.

Fig. S15. Microbial taxonomic variation after azithromycin administration in oral wash samples during 60 days’ follow-up. * P value≥0.05: there isn’t a significant difference in the relative abundance of the species at D14 compared to D0; P value< 0.05: there is a significant difference in the relative abundance of the species at D14 compared to D0.

Fig. S16. Sankey plots to describe the number of ZOTUs whose detection rate (A) and relative abundance (B) shifts or returns to the baseline level across the five timepoints in oral cavity microbiota. The shared ZOTUs whose detection rate (C) and relative abundance (D) had significant difference in oral wash samples (OW) microbiota and induced sputum samples (IS) microbiota.


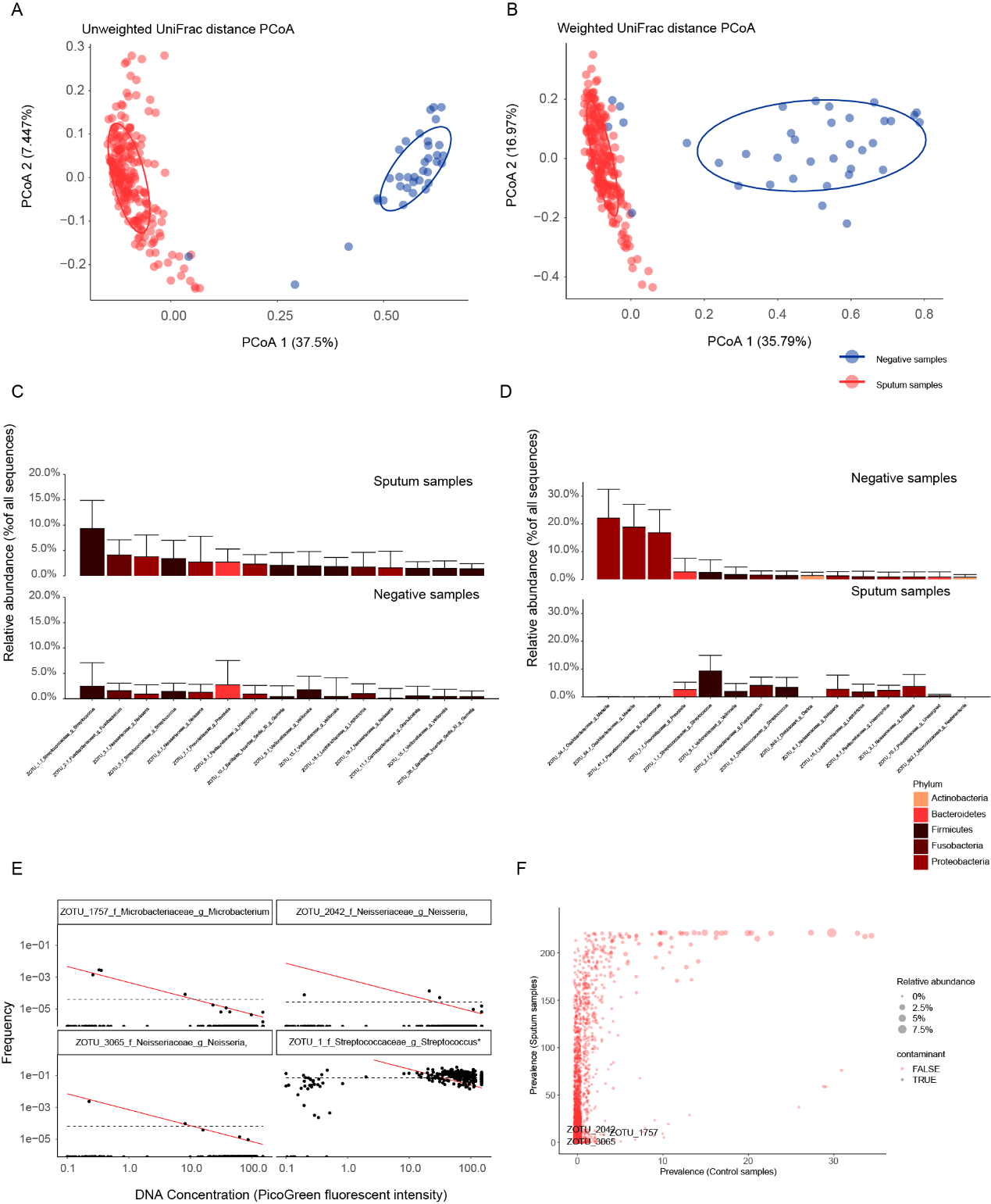


Fig. S1


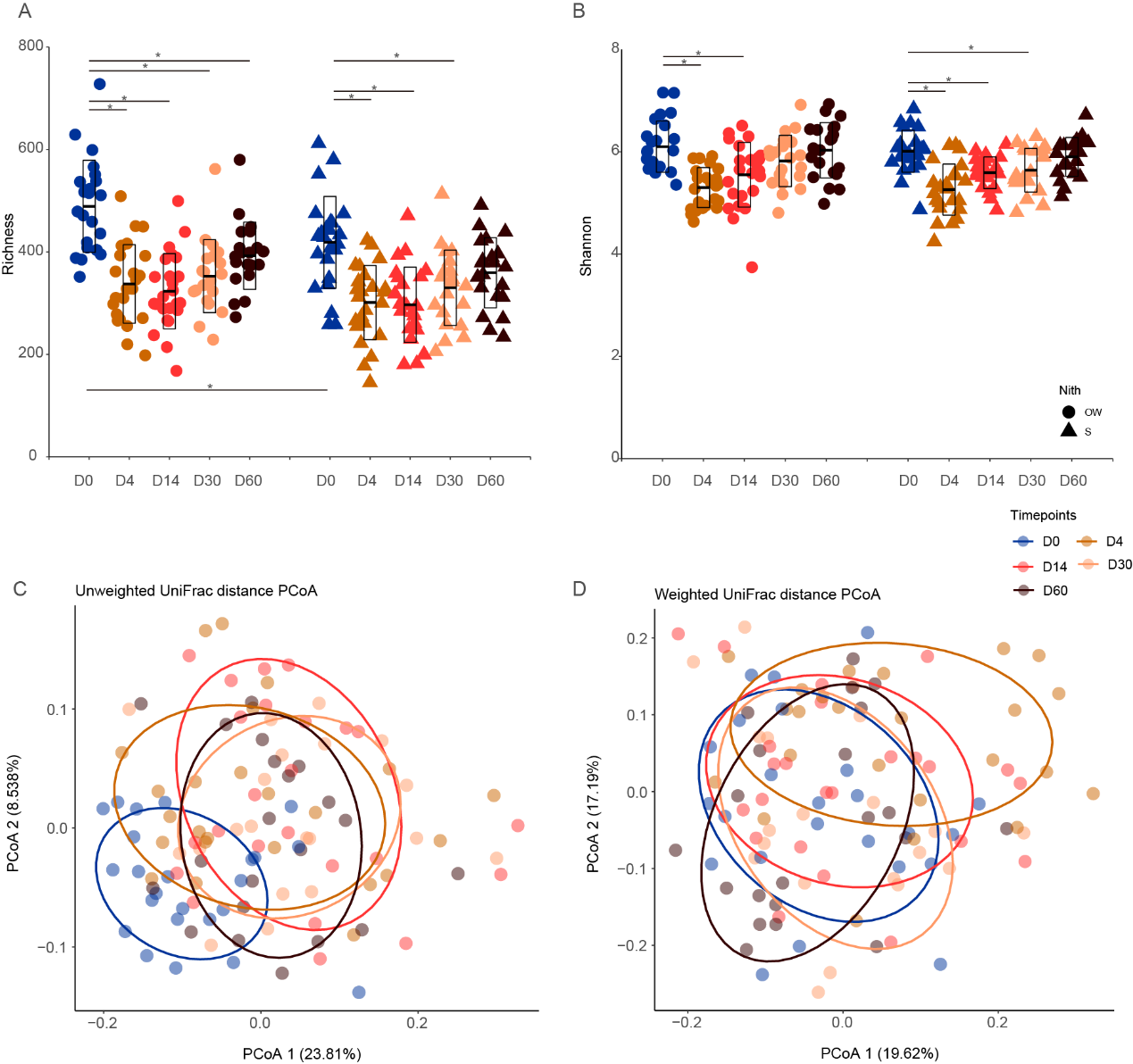


Fig. S2


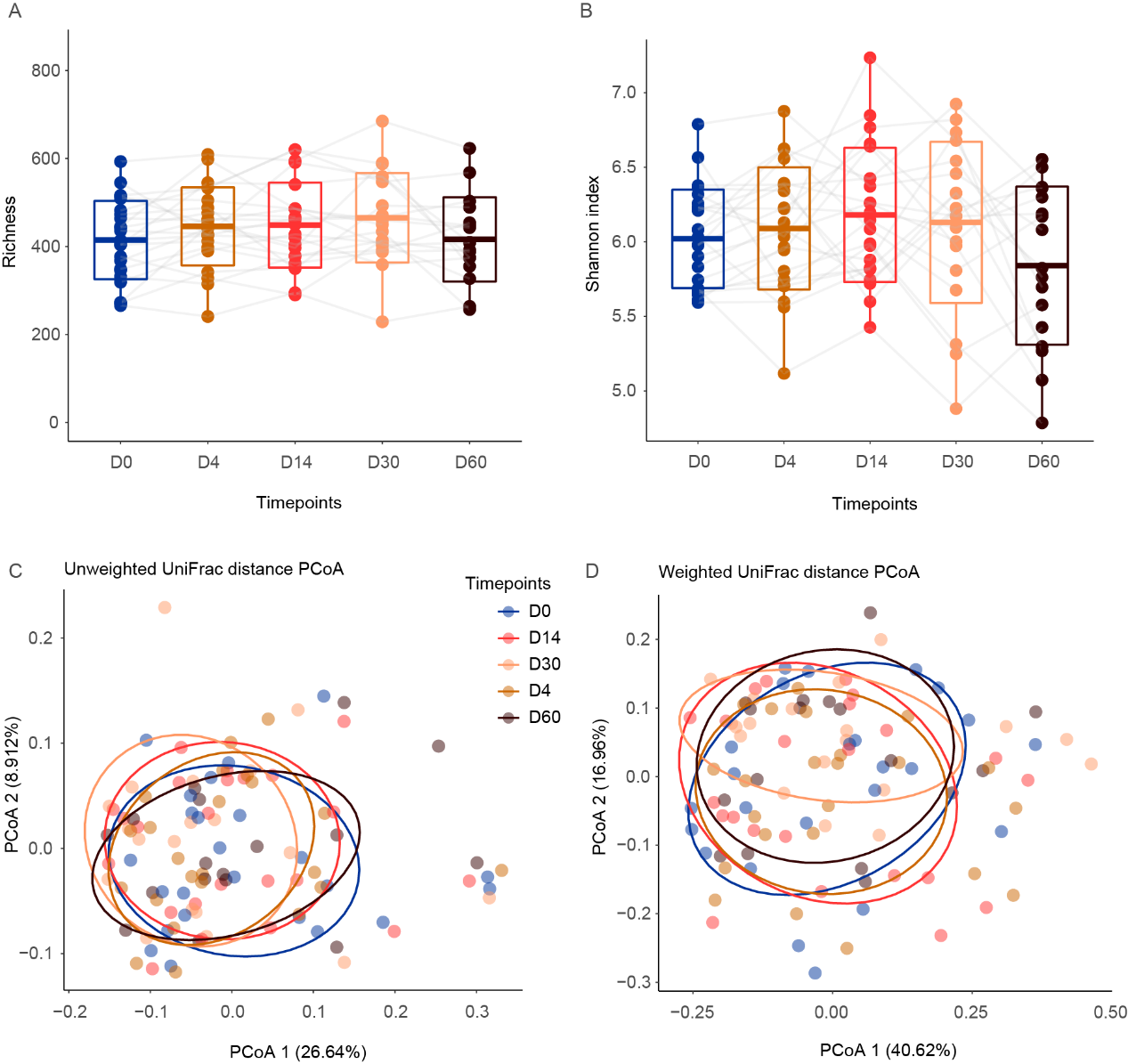


Fig. S3


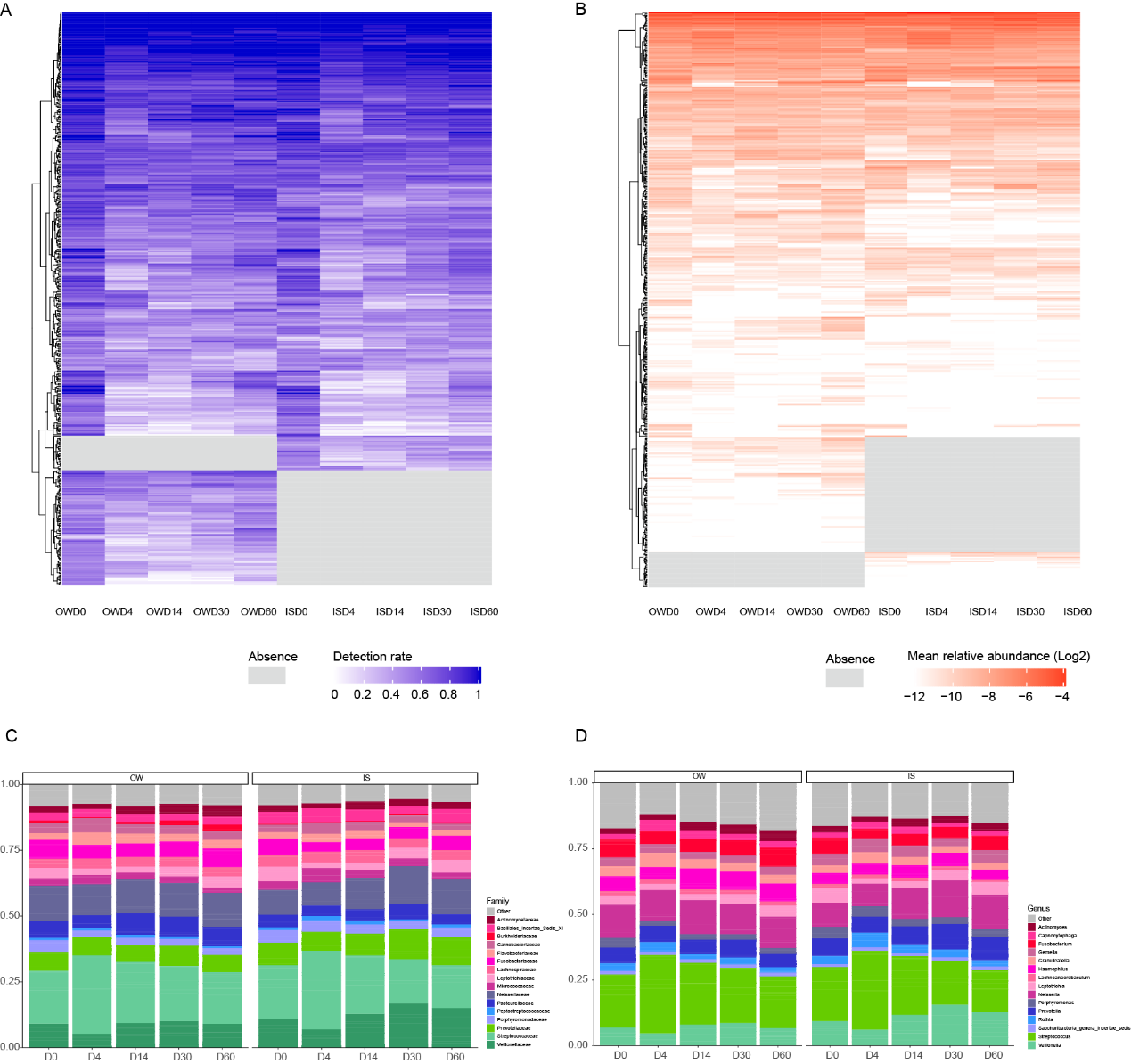


Fig. S4


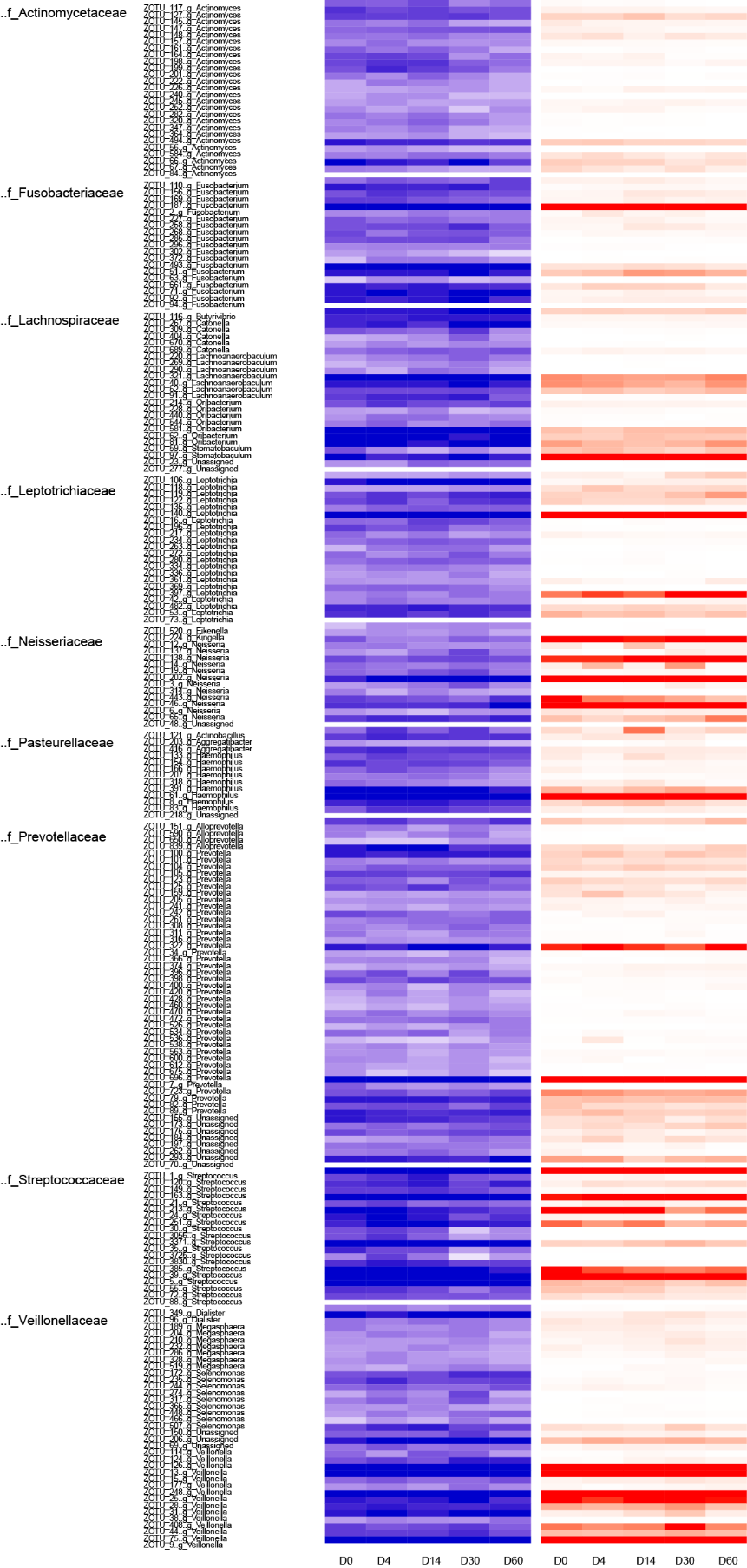


Fig. S5


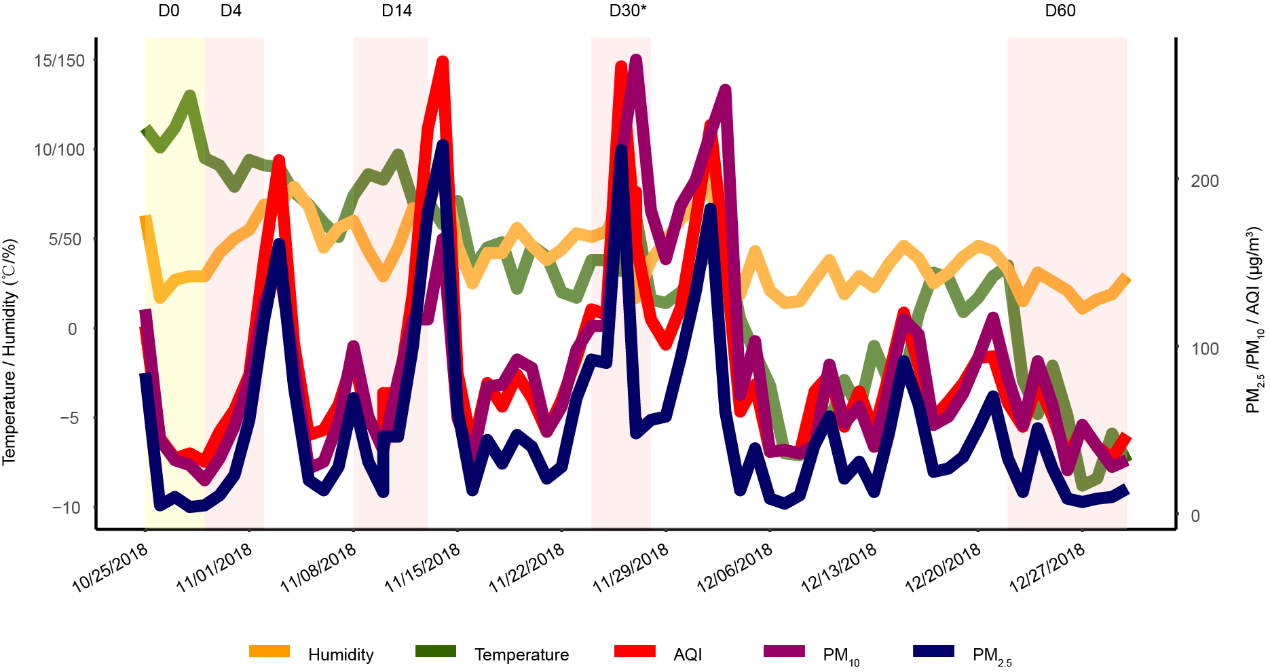


Fig. S6


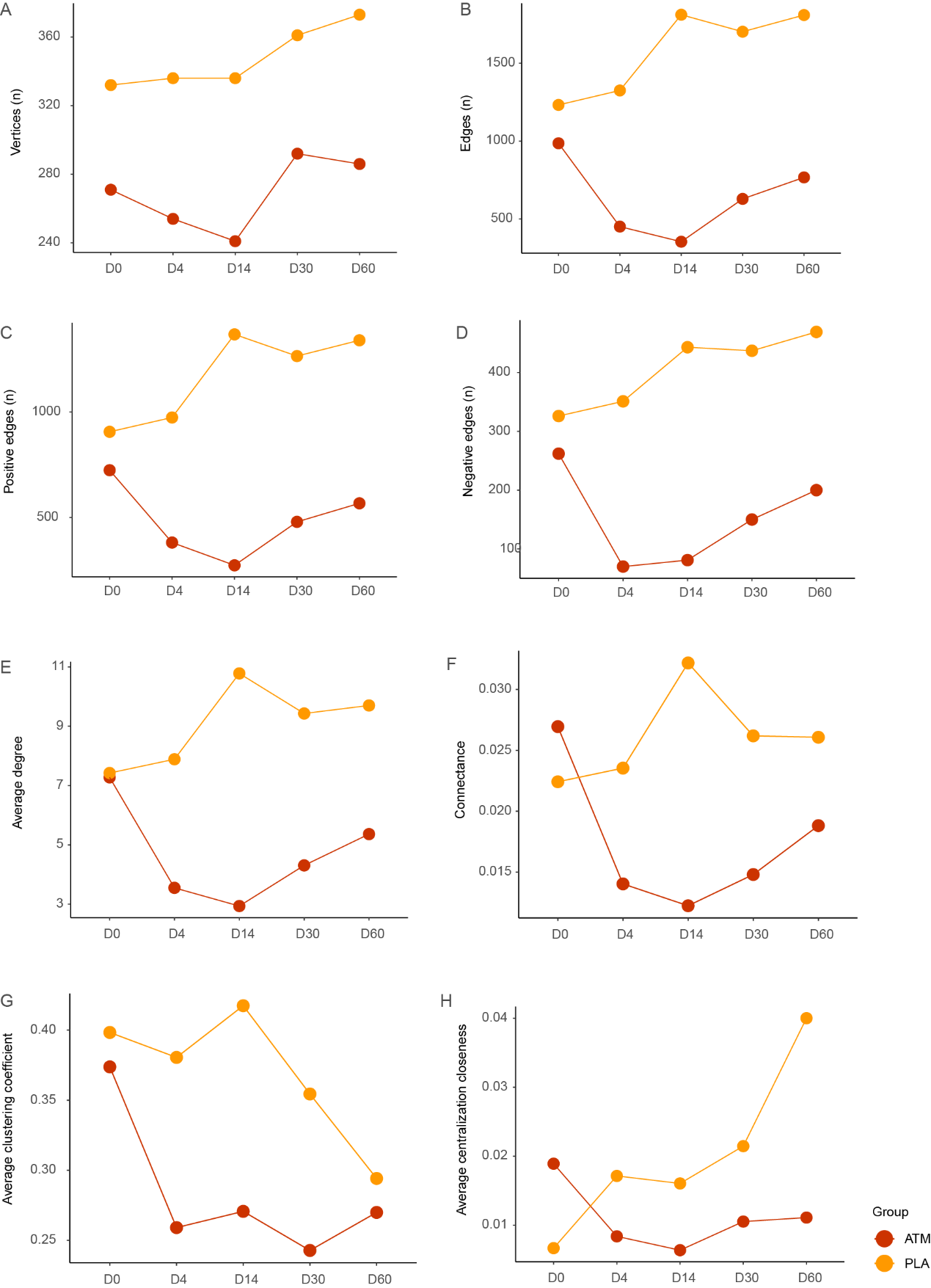


Fig. S7


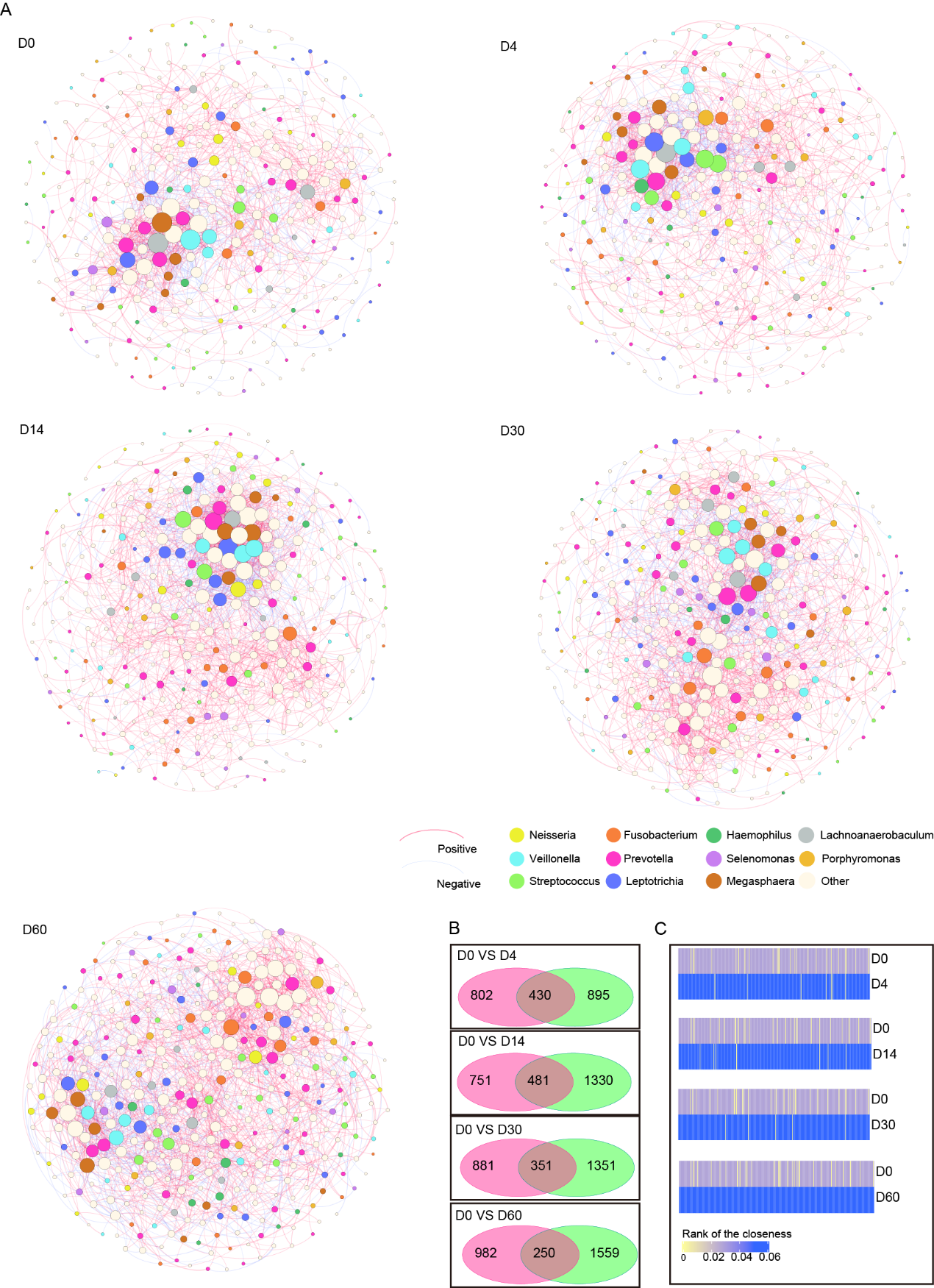


Fig. S8


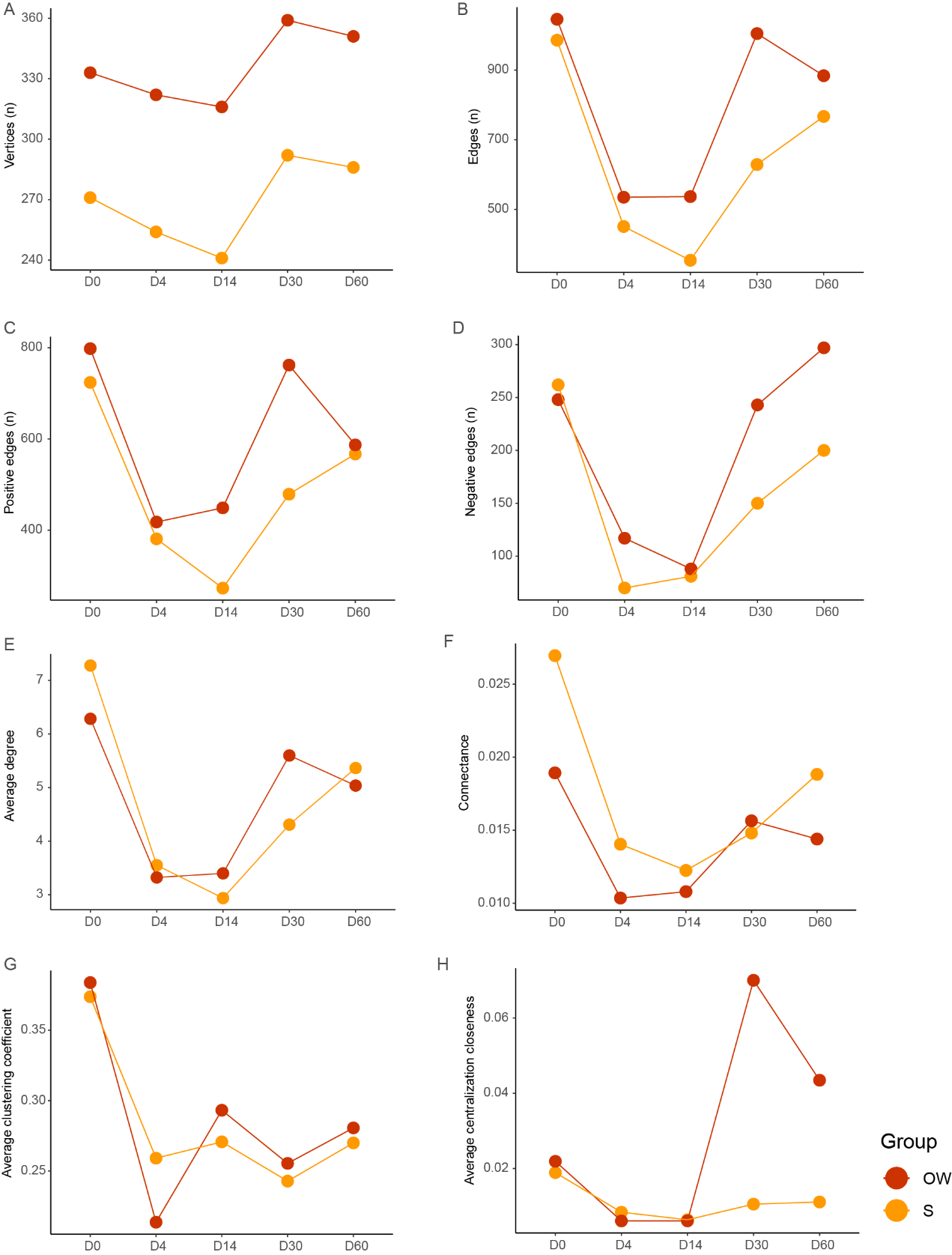


Fig. S9


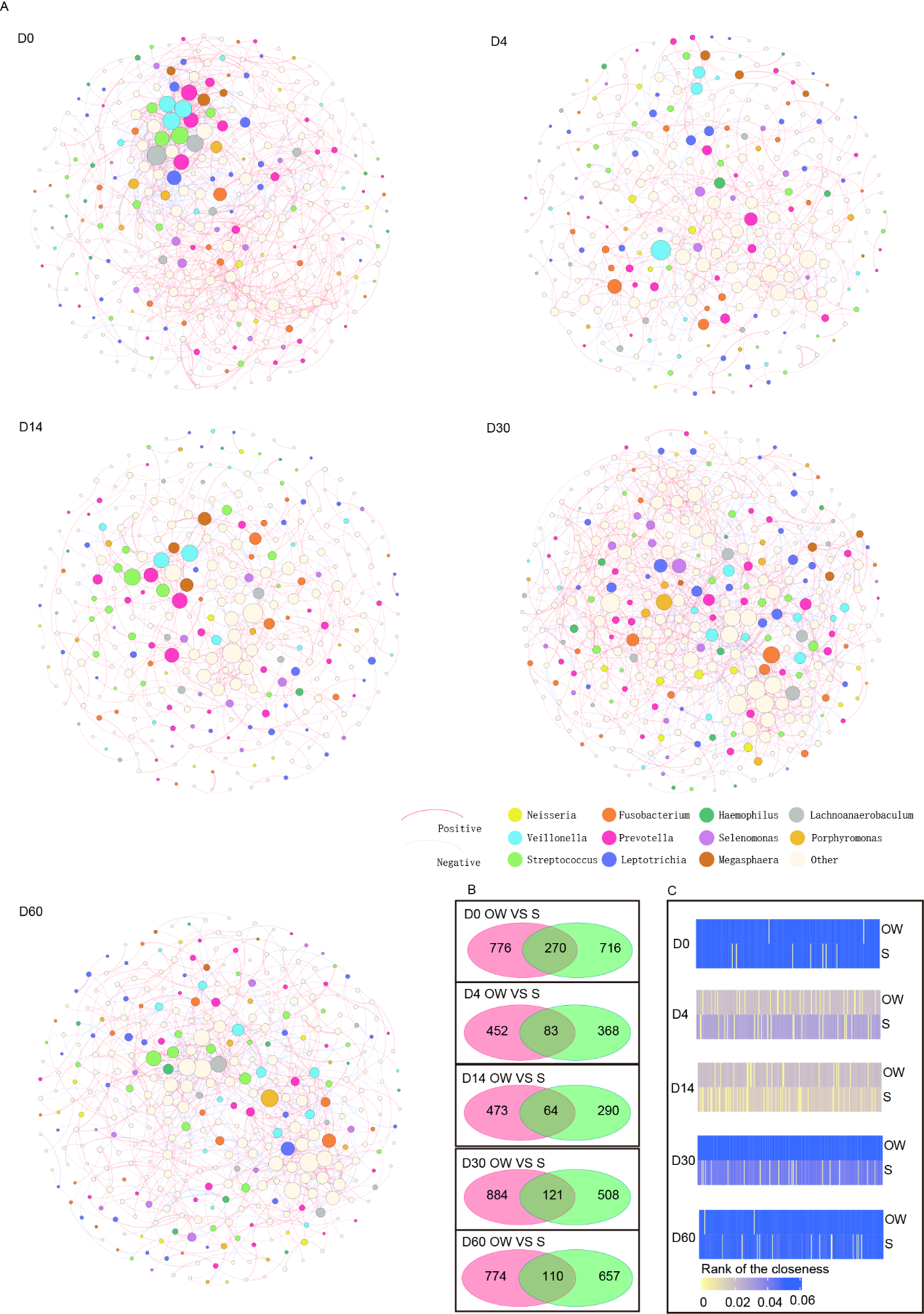


Fig. S10


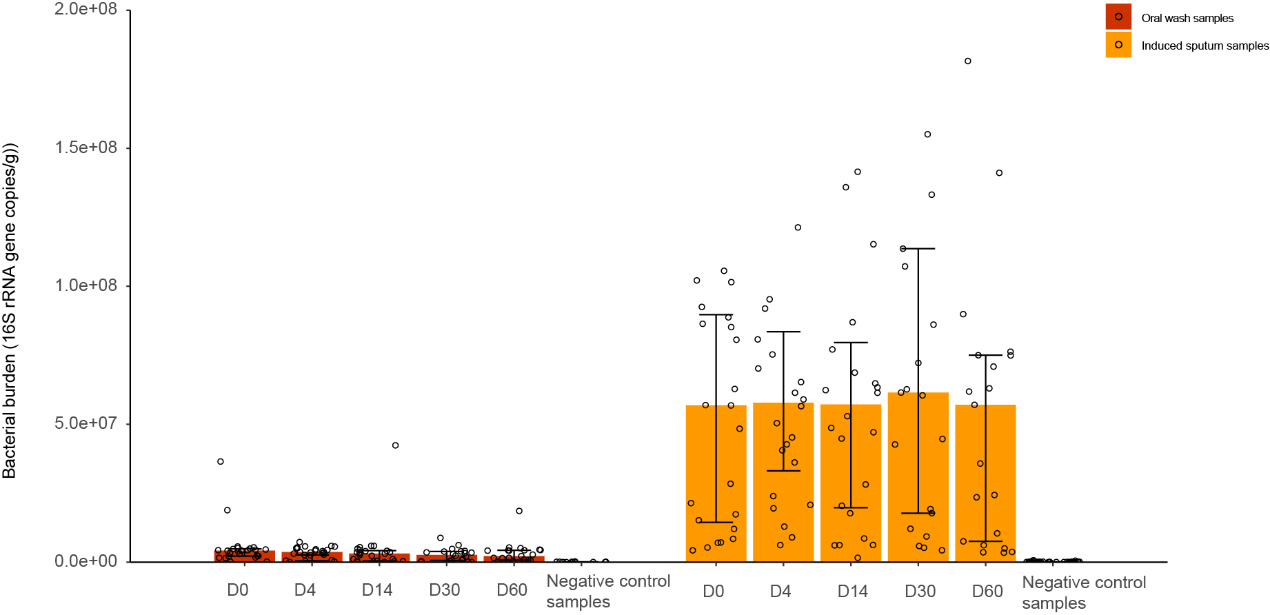


Fig. S11


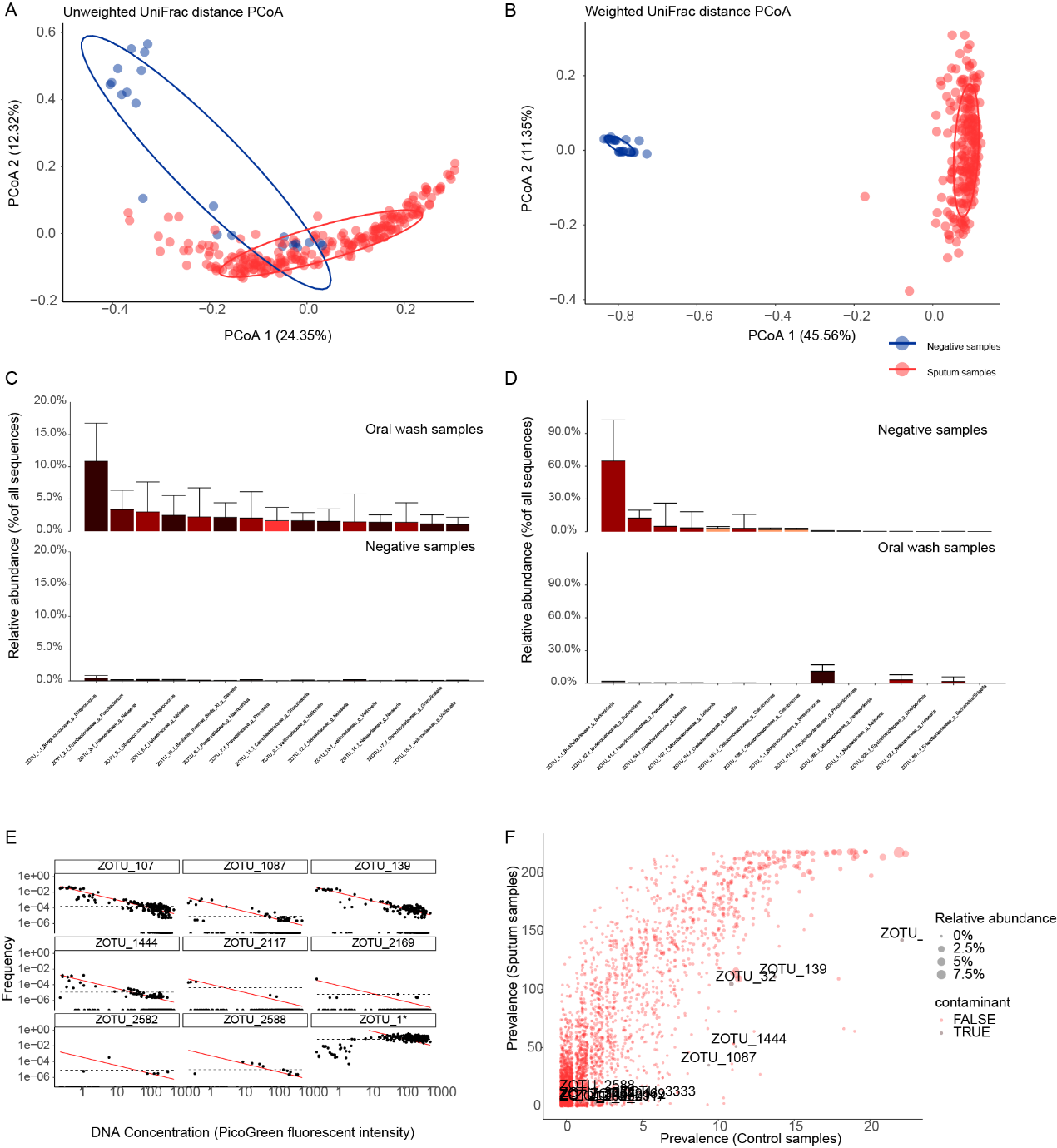


Fig. S12


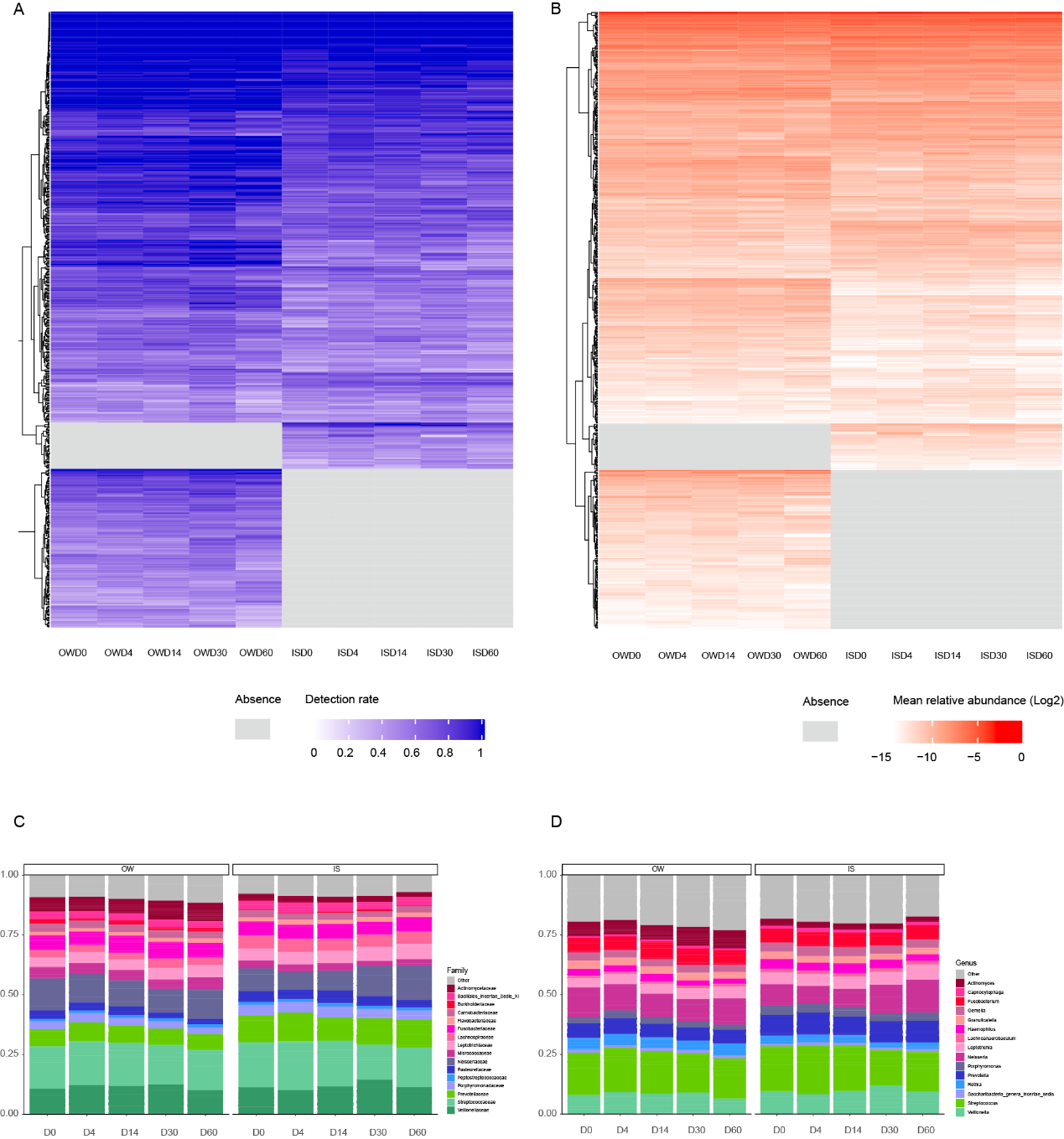


Fig. S13


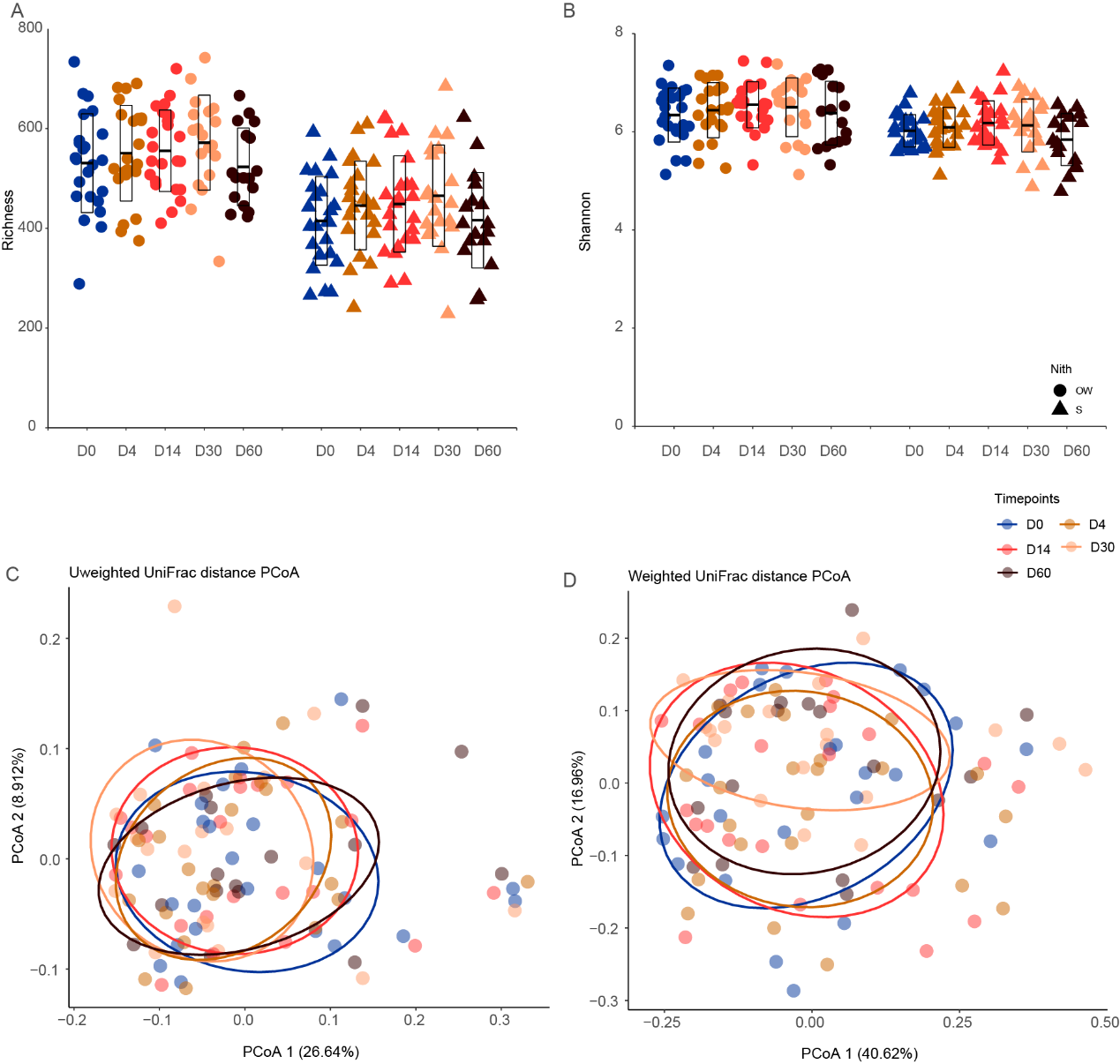


Fig. S14


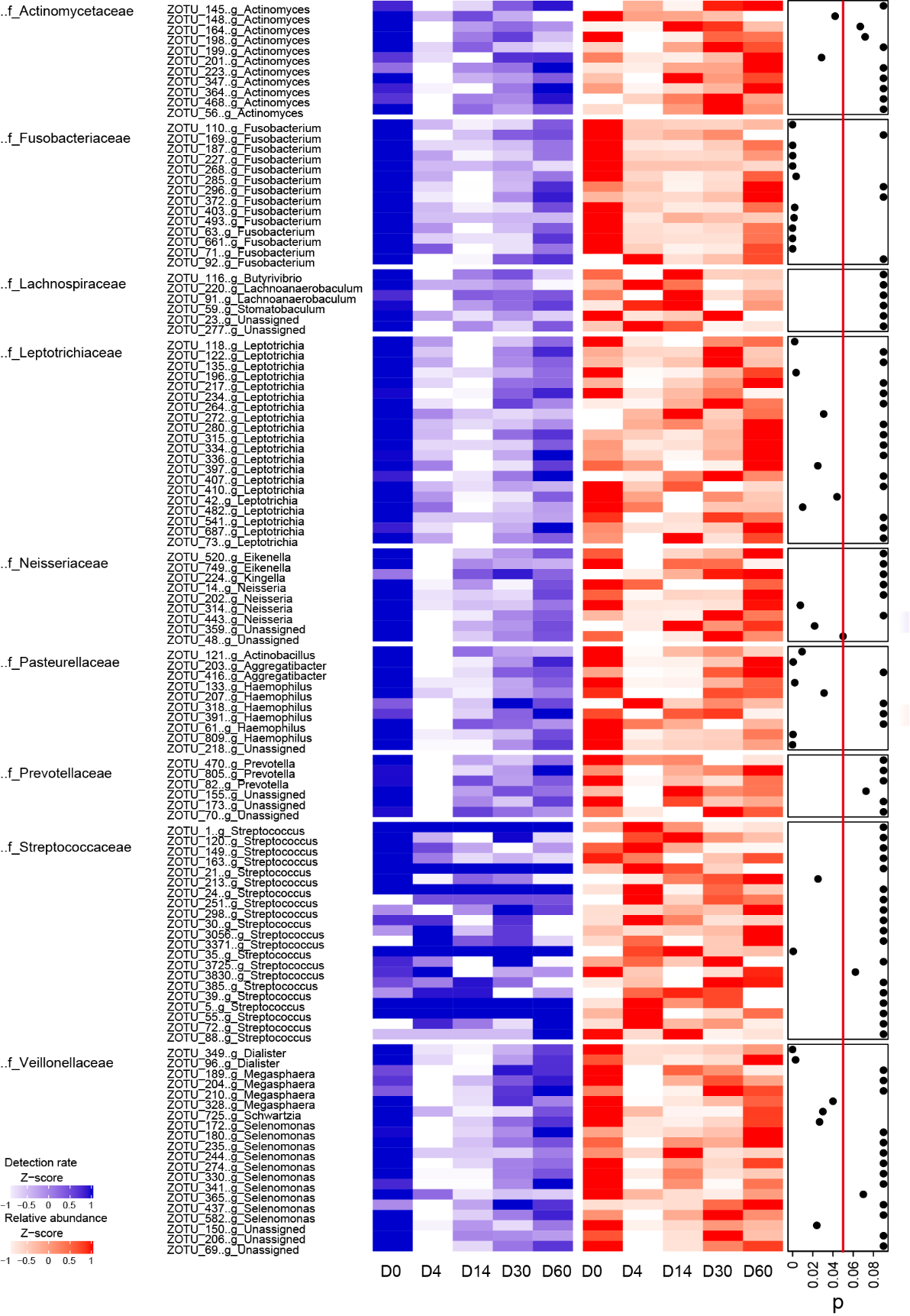


Fig. S15


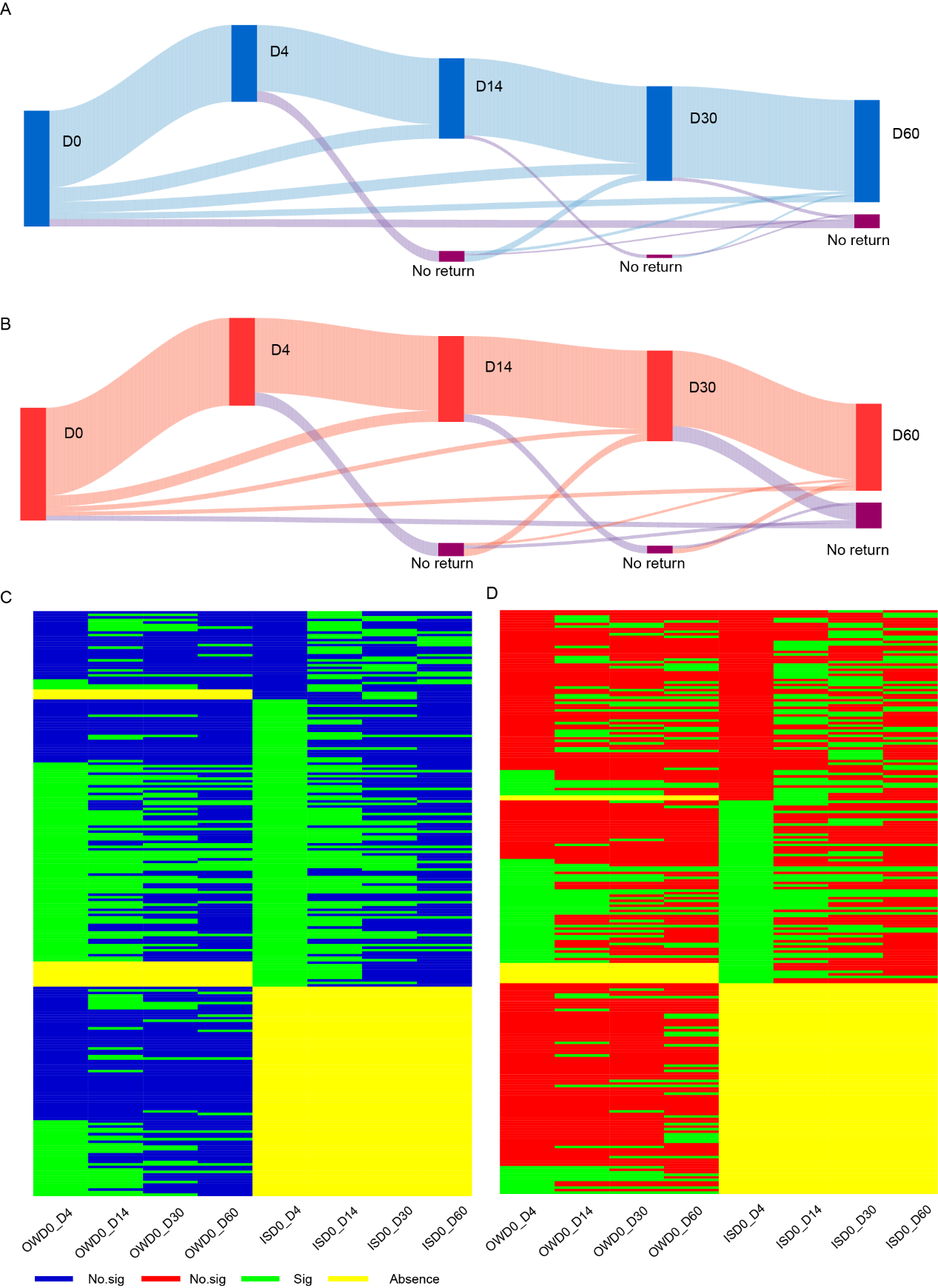


Fig. S16
